# The “Hidden Diversity” of Medicinal Plants in Northeastern Brazil: Diagnosis and Prospects for Conservation and Biological Prospecting

**DOI:** 10.1155/2013/102714

**Published:** 2013-10-20

**Authors:** Deyvson Rodrigues Cavalcanti, Ulysses Paulino Albuquerque

**Affiliations:** ^1^Laboratory of Applied Ethnobotany, Department of Biology, Federal Rural University of Pernambuco, Avenida Dom Manoel de Medeiros s/n, Dois Irmãos, 52171-900 Recife, PE, Brazil; ^2^State University of Alagoas, AL 115 Km 3, 57601-000 Palmeira dos Índios, AL, Brazil; ^3^Federal Institute of Education, Science and Tecnology of Alagoas, Avenida das Alagoas s/n, Palmeira de Fora, 57601-220 Palmeira dos Índios, AL, Brazil

## Abstract

Increases in ethnobotanical studies and knowledge in recent decades have led to a greater and more accurate interpretation of the overall patterns related to the use of medicinal plants, allowing for a clear identification of some ecological and cultural phenomena. “Hidden diversity” of medicinal plants refers in the present study to the existence of several species of medicinal plants known by the same vernacular name in a given region. Although this phenomenon has previously been observed in a localized and sporadic manner, its full dimensions have not yet been established. In the present study, we sought to assess the hidden diversity of medicinal plants in northeastern Brazil based on the ethnospecies catalogued by local studies. The results indicate that there are an average of at least 2.78 different species per cataloged ethnospecies in the region. Phylogenetic proximity and its attendant morphological similarity favor the interchangeable use of these species, resulting in serious ecological and sanitary implications as well as a wide range of options for conservation and bioprospecting.

## 1. Introduction

Medicinal plants are freely circulated in Brazil, particularly in informal trade settings where several types of plants are marketed for a wide range of illnesses (see [1]). Limited access to specialty medicine and an increasing interest in the so-called natural treatments account for the rapid increase of the trade in such products in Brazil [2]. 

The most important vendors of medicinal plants are located in urban centers, namely, in fairs and public markets, where consumers have easy access to a wide variety of medicinal plant species together with the corresponding therapeutic indications [3]. More specifically, the regional public markets act as spaces representative of the cultural production and biological diversity of a given area [1, 4] and as centers where the empirical knowledge retained in different areas and with different origins is aggregated, conserved, and spread. Thus, the regional public markets are the pillars of a complex, open, and dynamic system of knowledge [1]. 

Although promising for the biological prospecting of novel drugs and pharmaceutical products, actual research at such markets has some limitations, as the identity of the vast majority of the plant species traded there cannot be safely established by means of conventional methods [1, 5–7].

In contrast with community-based ethnobotanical surveys, where the investigated resources are directly accessible *in loco* [8–11], research at markets and fairs is much more complex, as a significant proportion of the plant products offered to the consumers are uncharacteristic or lack the elements required for accurate taxonomic identification (see [1, 5, 6]). As a rule, only parts of the plants are sold, to wit, the ones allegedly containing the active therapeutic components, such as barks, roots, seeds, flowers, and leaves, which are sometimes dehydrated, chopped, and/or ground. As a result, it becomes quite easy to mix or mistake a similar species with or for another. 

Several authors have previously expressed such concerns and proposed some methodological solutions to the problem [1, 12]. Various palliative techniques have been suggested for cataloging all medicinal plants available to the consumers at public markets, some of which are quite specialized and expensive [13–15], whereas others are feasible but not always viable [16, 17].

In addition to morphological similarities between species, another factor that makes it difficult to interpret the ethnobotanical data collected at public markets is the fact that multiple plants species are frequently known by the same vernacular name. Such events of semantic correspondence in ethnobotanical studies were initially detected by several authors [18–21] and then properly systematized by Berlin [22] in a study that sought to determine the relationships between the biological and traditional classification systems, thus establishing the grounds of ethnotaxonomy. 

Within that ethnotaxonomic approach, Berlin [22, 23] established the notion of underdifferentiation to define the semantic correspondence between different species that share a vernacular name, of which two types were described. Underdifferentiation type 1 occurs when the species involved belong to the same genus, and type 2 occurs when they belong to different genera. When only one species corresponds to a given ethnospecies, correspondence is defined as one-to-one or biunivocal [22, 23]. Several studies of local communities have employed these notions to identify similar patterns of semantic correspondence between different species [24–28]. The species subjected to underdifferentiation have been termed ethnohomonyms.

Although quite well adjusted to the local systems, such correspondences tend to overlap and become complex when different cultural origins become somehow intertwined [29, 30]. The overlapping of homonym ethnospecies makes the understanding of ethnobotanical data originating in environments where complex cultural networks are established even more difficult, as is the case with ethnobotanical studies at regional public markets (see [6]).

We define here the “hidden diversity” of medicinal plants as the set of different homonym ethnospecies “hidden” under the same vernacular name. We coined the term “hidden diversity” based on the analogy with the notion of a “hidden harvest,” which denotes the progressive and unofficially documented appropriation of the plant biodiversity in a given area [31, 32].

According to Krog et al. [6] the impossibility of distinguishing among homonym ethnospecies is one of the major limitations to the advancement of ethnobotanical research in public markets, particularly in the case of ethnopharmacological studies of plant conservation and bioprospection. Although that phenomenon has previously been detected in a localized and sporadic manner, its full dimensions have not yet been established. 

It is safe to assume that in Brazil, as a function of the plant biodiversity, environmental diversity, and multicultural and ethnic composition of the country [33], the number of homonym ethnospecies and consequently also the phenomenon of hidden diversity of medicinal plants is much more comprehensive and significant than suggested from the few occurrences recorded in the scientific literature. In the present study, we sought (1) to measure the hidden diversity, that is, the number of medicinal plant species subsumed under the same common name in the Brazilian northeast region; (2) to establish the different types of underdifferentiation of homonym ethnospecies; and (3) to assess the influence of biological diversity on the number of homonym ethnospecies. Finally, we sought to indicate some of the possible implications for conservation and biological prospecting. 

Assuming that the variety of homonym ethnospecies in a given region depends on the region's biodiversity, one might expect the following: (1) for the variation in the number of homonym ethnospecies to be directly proportional to the size of the sampled area, as larger areas theoretically include a wider variety of environments, and consequently, also greater biological diversity and (2) that a significant number of the homonym ethnospecies should be representative of the native flora compared to the group of species with one-to-one correspondence. 

## 2. Materials and Methods

### 2.1. Characterization of the Study Area

The northeast region of Brazil includes nine federal units and represents a total area of 1,558,196 km^2^, which corresponds to 18% of the country's territory. It is located in an intertropical zone limited by the Atlantic Ocean to the east and north, the Amazonian rainforest to the northwest, and the Cerrado (Brazilian savannah) domain to the west and southwest [34]. The vegetation is mainly xerophytic, being the Caatinga (Brazilian xeric shrubland), a highly peculiar biome with a high degree of endemism [35–37]. Atlantic ombrophilous forest predominates in the coastal area. Currently, this forest is one of the most seriously threatened biomes in the world, and only 5% of its original area remains [38, 39]. Enclaves of Cerrado and rainforest are widely present as areas of disjunct vegetation [40–43], making the Brazilian northeast region a strategic area from the perspective of global richness and biological diversity [44, 45]. 

From the demographic point of view, the total population of the northeast region comprises approximately 49 million inhabitants, primarily distributed along the coastal area where most state capitals and major cities are located, which together host approximately 40% of the population [34]. The cultural diversity of the northeast region is high due to the ethnic miscegenation resulting from the colonization of Brazil [46, 47], and the population includes Europeans, mostly Portuguese and Dutch, black slaves from Africa, and the various indigenous peoples. In addition, it is worth observing that in the last ten years, the economic growth of the region was significantly higher than the national average [34]. 

### 2.2. Data Survey

Six of the nine northeastern states were included in the analysis based on the need to survey the widest possible diversity of cultural representations and environments and the need to take into account the logistics of access and permanence at the study sites. For the purposes of the present study, we assumed that the expression of the regional culture is more diversified at the state capitals because they exhibit the largest population density, including immigrants from other states and/or the inland cities.

The states and corresponding capitals sampled were as follows: Maranhão/São Luiz, Ceará/Fortaleza, Paraíba/João Pessoa, Pernambuco/Recife, Alagoas/Maceió, and Sergipe/Aracaju. The primary site of medicinal plant trade in each state capital was identified, and thus the following markets were selected: the Mercado Central (Central Market) in São Luiz/MA, Mercado de São Sebastião (St. Sebastian Market) in Fortaleza/CE, Mercado Central in João Pessoa/PB, Mercado São José (St. Joseph Market) in Recife/PE, Mercado da Produção (Production Market) in Maceió/AL, and Mercado Albano Franco (Albano Franco Market) in Aracaju/SE.

Following an initial exploratory visit, an appointment was made for data collection. The plant vendors at each selected market were informed as to the nature of the study and invited to participate. Some vendors refused immediately, and others initially agreed and then went back on their original agreement. As a result, a total of 22 respondents were interviewed and provided a representative sample of the vernacular names of the plants traded in the region. In the state of Pernambuco, the ethnobotanical studies in public markets are already more advanced. Albuquerque and colleagues [1] previously found a significant decrease in the availability of plant vendors in this state based on only two samples obtained over an eight-year period. The *in situ* observations and data collected for the present study suggest that this decrease in availability may represent a general trend that can be explained by several factors. For instance, the lack of regulation and control of the sector in regards to health and ecological aspects may generate mistrust and insecurity among vendors. The vendors may also experience a lack of return research or “benefits" that would otherwise entice them to be informants. In addition, the harsh economic conditions of the country have removed a significant number of vendors from the market, and unrelenting derogatory campaigns have undermined the informal trade markets in the media. Vendors in the informal trade markets also experience increasing competition with food stores, which are common in large urban centers and usually have better infrastructure, availability, and sanitary conditions. There is also a lack of interest in new generations to continue the family traditions of using and trading medicinal plants.

After the study was explained, the respondents freely signed an informed consent form. The study was approved by the Research Ethics Committee of the Federal University of Pernambuco (Universidade Federal de Pernambuco—UFPE) no. 0039.0.172.0000-10, FR (Folha de Rosto—Title Page) 3139660. 

Although some authors [1] have reported that several terms are used to describe vendors of medicinal plants, eventually including hierarchical criteria, in the present study, we used the generic term “herbalist” (locally known as “erveiro”) to allude to any type of vendor of medicinal plants. The term ethnospecies is used in the present study to allude to the common or vernacular names given to the medicinal plants. 

Using a field notebook, we made records of the catalogs of plants traded by the herbalists as mentioned in semistructured interviews [48]. For the purposes of the study, the plants available in stock at the time of the study as well as those traded in the previous 12 months were taken into consideration. The common names of the plants were recorded as spelled by the respondents. 

### 2.3. Data Analysis

The ethnobotanical data supplied by the herbalists in the various studied northeastern states were transcribed and entered in digital spreadsheets using MS Excel 2003 software, thus creating a Market Relational Database (MRD). The MRD was used to map the geographical distributions of the ethnospecies across the Brazilian northeast region and identify the most frequently occurring ones. 

 In parallel, an Ethnobotanical Survey Database (ESD) was created and populated. For that purpose, 55 ethnobotanical surveys of the northeastern states were identified, and the listed species and ethnospecies were entered in the ESD. The plants not identified at the species level were not included. Only relevant studies were selected: most (45) were published in major scientific journals, seven were Master's dissertations, one was a doctoral thesis, one a book, and one the Development Plan of a major Brazilian university (Federal University of Bahia—Universidade Federal da Bahia, UFBA).

 The data entered in both databases (MRD and ESD) were then crosschecked to produce a detailed list of the ethnospecies mentioned both in the ethnobotanical surveys and by the respondents in our study, with the corresponding species. This step allowed for the identification of the homonym species and their clustering around the corresponding ethnospecies. 

 We selected a sample corresponding to 40% of the ethnospecies included in both databases (MRD and ESD) based on their frequency in the ethnobotanical surveys. Thus, only the 165 most frequent ethnospecies out of a total of 406 listed in the ethnobotanical surveys were selected for analysis.

 The sampling criteria used were based on two assumptions: (1) ethnobotanical research is still incipient in most of the northeast region, and thus, infrequent ethnospecies might suggest a merely temporary pattern of semantic correspondence, consequently masking the results, the number of one-to-one correspondences in particular and (2) the ethnospecies most frequently mentioned in the regional ethnobotanical surveys might represent the patterns of semantic correspondence in a more unequivocal and reliable manner. 

 The corresponding species were allocated to three groups: one comprised the species with one-to-one correspondences, the second, the homonym ethnospecies with type 1 underdifferentiation, and the third, the homonym ethnospecies with type 2 underdifferentiation, according to Berlin's [23] classification. The corresponding species were subjected to synonym analysis; the names that are currently valid were duly recorded based on the List of Species of the Brazilian Flora 2012 [49] and the database of the Missouri Botanical Garden [50], which were also used to establish the biogeographic status of each species to classify them as native or exotic. 

 To assess whether underdifferentiation (*sensu* Berlin [23]), expressed as the number of homonym ethnospecies, varies as a function of the biological diversity of a given area, we compared the results corresponding to the northeast region with a geographically narrower sample, based on the assumption that the larger the area, the wider the environmental variety, and thus, the more diversified the flora. 

 That narrower sample was represented by the state of Pernambuco, which is the northeastern state most thoroughly studied from an ethnobotanical perspective. The numbers of homonym ethnospecies and one-to-one correspondences of the northeast region were compared to those of Pernambuco. The frequency of species in the respective categories of semantic correspondence (i.e., one-to-one and underdifferentiation) was analyzed by means of *G* tests [51] as were the percentages of native and exotic species in each group. 

## 3. Results

The ethnospecies (*n* = 165) sampled based on the data collected at the visited markets exhibited correspondence with 459 species, corresponding to 228 genera and 90 families (Table 1). The ratio of species to ethnospecies was 2.78. From the total number of analyzed ethnospecies, only 41 (25%) exhibited one-to-one correspondence, whereas 124 (75%) exhibited underdifferentiation and correspondence to 418 species. Approximately 62% of the homonym ethnospecies exhibited two or three corresponding species, although in some cases, a single ethnospecies included up to nine corresponding homonym species, as, for example, “quebra-pedra” (stonebreaker) (Table 1). 

Analysis of the data corresponding to the state of Pernambuco alone identified 138 out of the 165 ethnospecies found in the northeast region, which exhibited correspondence with 203 species. The ratio of species to ethnospecies was 1.46. The pattern of correspondence included 89 (64%) instances of one-to-one correspondence and 49 (36%) of underdifferentiation; the homonym ethnospecies represented a total of 114 species. 

**Figure 1:**
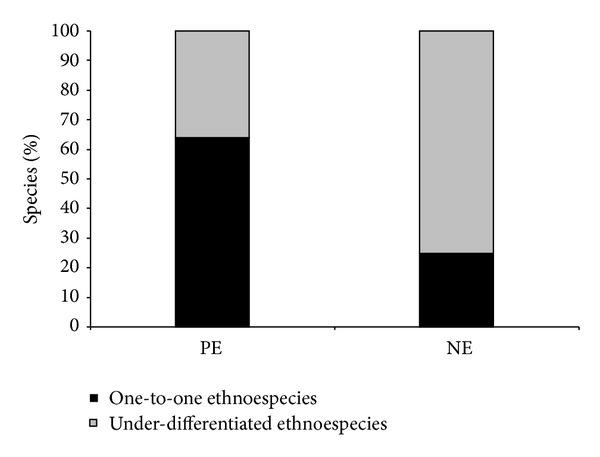
Percentages of ethnospecies that exhibited one-to-one correspondence and underdifferentiation marketing in the northeast region and the state of Pernambuco, Brazil.

Comparison of the data from the state of Pernambuco and the northeast region showed variation in the number of one-to-one correspondences that was inversely proportional to the size of the sampled area, whereas the number of homonym ethnospecies varied in proportion to the size of the sampled area, as shown in Figure 1. Consequently, the homonym ethnospecies predominated in the northeast (NE) sample (*G* = 48.41; df = 1; *P* < 0.00001). 

**Figure 2:**
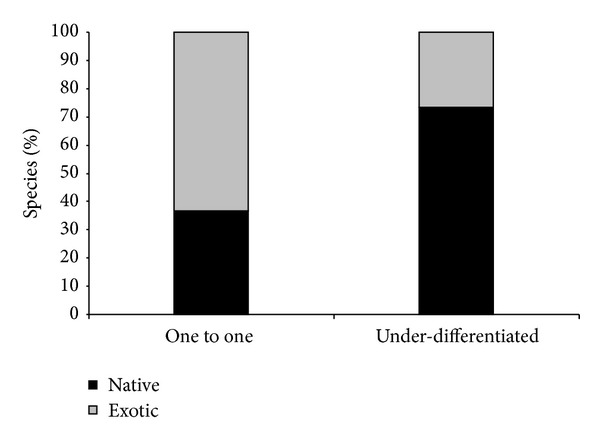
Percentages of native and exotic species in the group of ethnospecies that exhibited one-to-one correspondence and underdifferentiation marketing in the northeast region and the state of Pernambuco, Brazil.

In the group of homonym ethnospecies, 309 (74%) were representative of the native flora, and 109 (26%) were exotic species. In the group of ethnospecies with one-to-one correspondence, 15 (37%) were representative of the native flora and 26 (63%) were exotic species (Figure 2). The proportion of native species relative to the proportion of exotic species was therefore significantly greater for the under-differentiated ethnospecies compared to the one-to-one ethnospecies (*G* = 22.52; df = 1; *P* < 0.00001). 

Among the 418 homonym ethnospecies, 256 (61.3%) were congeneric (type 1 underdifferentiation), and 77 (18.4%) exhibited correspondence at the genus level only (type 2 underdifferentiation). That is to say, 61% of the species bear correspondence to at least one other species of the same genus with the same vernacular name, whereas 18.4% of the homonym ethnospecies exhibited correspondence with one or more species belonging to other genera in the same family. In some cases (20.3%), the homonym ethnospecies belonged to different families, such as the ethnospecies “fedegoso” (coffee senna) and “capeba” (cow-foot leaf) (Table 1). 

## 4. Discussion 

### 4.1. Hidden Diversity in Regional Markets

Knowledge of the hidden diversity of medicinal plant species represents an important tool because it might point to the possible patterns of substitution of homonym ethnospecies in a given area. In the case of northeast Brazil, 75% of the plants traded in regional public markets exhibit correspondence with more than one plant species.

As most (74%) such species are representative of the native flora, we might infer that the regional markets are largely supplied by natural stocks. Because the demand for medicinal plants is continuously increasing [2], the gradual exhaustion or scarcity of resources might make the substitution of homonym ethnospecies unavoidable and increasingly more frequent, particularly in the large cities where 70% of the population resides [34] and where access to medicinal plants is primarily mediated by commerce. 

Precisely for that reason, it is safe to assert that semantic plurality is manifested most frequently at the public markets of large cities, which are privileged spaces where significant amounts of people, products, and knowledge circulate on a daily basis. Thus, such markets afford an extremely favorable scenario for comparative ethnobotanical studies at a regional level. 

In recent years, ethnobotanical research in regional public markets has provided an important platform for conservation studies and biological prospecting. However, the limitations to species identification represent the major hindrance to the growth of research in such locations [6] as well as to the assessment of hidden diversity and events of homonym ethnospecies substitution, as most of the plants are sold in parts or pieces that are sometimes completely uncharacteristic. 

For that reason, homonym ethnospecies go easily unnoticed when commercial medicinal species are cataloged, the more so the more remarkable the morphological similarities are. In this regard, 61.3% of the hidden diversity of the medicinal plants of the northeast region is congeneric, that is, exhibits type 1 underdifferentiation, which denotes phylogenetic proximity and consequently morphological similarity [102]. This similarity makes the understanding of the ethnobotanical data collected at public markets even more difficult. 

To prevent this situation, the criteria adopted for the identification of species by some studies conducted in regional public markets are based on the vernacular nomenclature, sometimes as a complementary identifier [12, 103] and other times as the primary criterion [6]. In places where the catalog of medicinal plants and the data relative to their biodiversity are comprehensive, common names might possibly be used quite safely. However, this is definitely not the case in Brazil, where the repertoire of medicinal plants in these marketing spaces is largely a hidden diversity. 

Additionally, due to the explicit difficulty of recognizing and identifying the plant species in public markets and the progressive increase in the substitution of homonym ethnospecies, the vulnerability of consumers tends to become more serious, and possible risks related to safety and efficacy might be potentiated when one species is indistinctly replaced with another. This phenomenon occurs because most of the Brazilian medicinal plant species have not yet been subjected to appropriate studies that would establish their use in a scientifically safe manner, so to speak, that is, describing their side effects, contraindications, toxicity, and effective therapeutic action. 

Because the only plant material available for species identification is that sold at the markets, whereas the harvesting sites are usually inaccessible due to their distance or the unavailability or mistrust of the harvesters—as a large part of harvesting is indiscriminate—the resolution of this impasse necessarily demands more specialized taxonomic procedures, such as micrography and molecular taxonomy. 

In this regard, several techniques have been widely applied to the resolution of this type of taxonomic problem [14, 15, 104–106], to support scientific research and as a tool for the surveillance and control of commercial plant and animal products. *Barcoding *is one of the most promising among such techniques and has already been applied to the identification of plant species in public markets [107]. This technique consists of the identification of species based on the differentiation of genetic sequences in specific DNA regions [108].

The use of molecular taxonomy might in time become a very important and practical tool for cataloging the hidden diversity in public markets and thus contribute to a better understanding of the biodiversity flow in a given area and, consequently, the frequency with which homonym ethnospecies are being interchangeably used in public markets. A reliable cataloging of this biodiversity affords multiple possibilities for further biological and cultural studies and must be considered as crucial for the advancement of ethnobotanical research. 

### 4.2. Implications for Conservation

From the perspective of conservation at the regional level, one should not ignore the hidden diversity of medicinal plants, as this diversity represents the possible variations in the range of species that are effectively used relative to the multiplicity of homonym ethnospecies and the biological diversity of a given area. On such grounds, one might infer that the larger the number of homonym ethnospecies, the higher the odds that the pressure of use is, or might eventually become, distributed among more than one plant population, as in our study where a significant number of native homonym ethnospecies (74%) was found.

 When, conversely, the frequency of use predominantly affects one species, the risks are patently greater for the species affected but also for others with the same vernacular name, as due to substitution, those others might become subjected to an intense and fast extractivist pressure that compromises their resilience, particularly in the case of the most vulnerable populations, leading to their collapse. 

The species *Myracrodruon urundeuva* provides a good example of the possible impact of the extractivist pressure on more than one plant population. In this case, another species, *Schinus terebinthifolius*, which is also native and belongs to the same family, is currently traded under the same generic name (“aroeira”—Brazilian peppertree) in the city of Recife [1]. Therefore, these species are interchangeably used, even though they belong to different genera, as the used parts do not allow for a clear differentiation. 

It is possible that such homonym ethnospecies are being overlapped in an unconscious and undocumented manner at the points of sale, especially in the case of populations that are no longer easily found in their natural reservoirs and that precisely for that reason are subjected to substitution processes. For example, the case of “espinheira santa” (*Maytenus ilicifolia*), which following its long-term indiscriminate harvesting became a threatened species [109] and is associated with several substitute species that currently occupy the same semantic-therapeutic niche [110].

This type of approach must be taken into account upon establishing conservation priorities and efficient management strategies, as accurate knowledge of the hidden diversity of medicinal plants and the possibilities for efficient exchanges among homonym ethnospecies might favor a more balanced distribution of the extractivist pressure, thus minimizing its impact, avoiding the collapse of populations and promoting greater resilience. 

The applicability of hidden substitutions of species to biological conservation is thus in keeping with explicative models related to the utilitarian redundancy hypothesis [52], according to which a larger number of species within one utilitarian category leads to greater mutual support and protection of the associated species as well as increased resilience.

Thus, we might assert that the phenomenon of the hidden diversity of medicinal plants gives support to utilitarian redundancy as an explanatory model for the pressure of use, as the overlapping species are subsumed under one and the same identity and consequently the same therapeutic indications, as their corresponding practical value is culturally well established. 

Because, based on the strength of tradition, the homonym ethnospecies are functional analogs, the remaining task is to distinguish each one of them and establish the level at which the preference for and/or access to each particular species occurs and then to define the degree of utilitarian redundancy, which is also hidden, so to speak. For that purpose, once again the elaboration of a taxonomically reliable record of this biodiversity is required. 

Within that context, the assessment of hidden and redundant biodiversity becomes an important predictive ecological tool, as a function of the perfect semantic-therapeutic juxtaposition of the homonym ethnospecies at the regional level. Public policies for the conservation, regulation, control, and use of medicinal plants in Brazil should not ignore the regional level and its implicit cultural and biological richness [111–113]. From this perspective, comparative ethnobotany will become an indispensable tool in decision making and actions aimed at the sustainable use of biodiversity. 

### 4.3. Implications for Bioprospecting

Several studies [114–116] have found similar biochemical compositions in related species, which might point to similar uses within the range of applications already well established by tradition. The biochemical constitutions of species in the same family quite commonly include the same pattern of secondary components [114]. 

 Nevertheless, the therapeutic efficacy and the risks associated with the use of the vast majority of species acknowledged as medicinal by the population have not yet been assessed [117]. With regard to the medicinal species whose safety and efficacy have been demonstrated, ethnobotanical studies that include their hidden diversity might contribute to the identification of more efficacious species as well as of those more promptly available for consumption. 

Therefore, the identification of homonym species with similar uses might not only reduce the pressure of use on the natural reservoirs but also allow for easier and more encompassing access for a larger number of people. In this regard, it is worth stressing that to be efficient, public policies addressing access to medicinal plants must take into consideration the natural distribution of the species, when it is spontaneous, and the limits of its ecophysiological tolerance, in the case of cultivated species. The identification of homonym species might represent an alternative in both cases. 

Recently, the Brazilian government published a list of 71 medicinal plant species recommended for use by the Unified Health System (Sistema Único de Saúde—SUS) [118]. As a function of the continental size of Brazil and its environmental diversity, the distributions of some of these medicinal species are not homogeneous across all regions. Species typical of the south and southeast regions are hardly found in the north and northeast regions, and vice versa. Therefore, in both cases, there are homonym ethnospecies occupying the same semantic-therapeutic niche of many species in the corresponding region.

The case of *Uncaria tomentosa* is exemplary. This plant, native to Amazonia (north region), is commonly known as “unha de gato” (cat's claw) and acknowledged for its remarkable anti-inflammatory activity. Although it was included in the SUS list, access to this plant is extremely restricted in other Brazilian regions, which, however, will not prevent the emergence of other types of “cat's claw.” There are at least six different species known as “cat's claw” in the northeast region alone, five of which are native and one subspontaneous, corresponding to four different families, thus denoting the generality of the common name and the particularity of the biological expression. 

According to Albuquerque and Hanazaki [119] one of the basic rules in biological prospecting is to identify the criteria used by people to select plants for medicinal use. According to those authors, the process underlying such selection might point to more efficacious strategies and shortcuts for the identification of key species relevant to bioprospecting. 

A preliminary assessment of the distributions of the ethnospecies in the present study indicated that several species, including exotic ones established centuries ago, have corresponding homonym ethnospecies from the local flora. This is the case for cinnamon, watercress, elder tree, artichoke, clove basil, plum, and rosemary, among others (see Table 1). Such correspondences were also found when medicinal plant species were compared with the names of drugs (generic and trademarks names) with widely acknowledged therapeutic effects, such as Meracilina, penicillin, Novalgina, aspirin, Terramycin, and ampicillin, among others [9, 11, 24, 93, 120, 121]. In such cases, the species is named after its corresponding drug name, thus representing a flagrant instance of classification based on analogical use. 

Similarly, based on the wide variety and distribution of homonym ethnospecies, we might infer that the development of knowledge at the local level seeks to subsume the available biodiversity under the already established and culturally consolidated semantic-therapeutic patterns. For that reason, when key species with high cultural relevance are absent, the communities tend to opt for species substitutions [52].

As a function of the existence of semantic-therapeutic niches and the impossibility of filling them with traditionally acknowledged species, an analogy-based local process appears to be triggered. According to the available data, several mechanisms of cultural selection are operative in this analogy-based local process, whereby the most fitting pieces of local knowledge become prevalent and amplified across the community, pointing to the locally accessible species, which thus come to be used as corresponding (homonym) ethnospecies. This hypothesis is corroborated by the high frequency of homonym ethnospecies representing the native flora (73%). 

Comparative ethnobotanical studies of different regions might eventually elucidate the possible role of vernacular names as models for the manifestation of the expression of local biodiversity or the measure and circumstances under which a peculiar regional classification system tends to prevail at the expense of allochthonous and/or general systems. In addition, the identification of the level of semantic similarity of species at the local level might contribute to a better understanding of the process of construction of local/regional knowledge and make the planning of the use, prospection, and conservation of these resources more efficient [122]. 

## 5. Conclusions

Regardless of being a frequent process, affecting either some or the full set of species of a given region, the substitution of homonym ethnospecies denotes a novel consumption option for a well-established cultural practice involving limited products within a commercial niche consolidated by tradition. For that reason, even where the level, frequency, and circumstances under which such substitutions occur might not be identified in the near future, some relevant questions have already been raised. Such questions, which might contribute to optimizing the use of medicinal plants in a safe and more sustainable manner, include the following. (1) How might the homonym species be alternately used for the same therapeutic action and how efficacious are they? (2) For which homonym ethnospecies might divergent uses, absence, or differences in the level of therapeutic efficacy be currently listed? (3) What are the health risks to people who, either travelling or at their original place of residence, indiscriminately consume different species subsumed under the same common name? (4) What tools might be created to support consumers and researchers in the understanding and interpretation of the semantic plurality associated with medicinal plants? (5) Which bioprospecting actions and management plans have taken the hidden diversity of species at the regional level into consideration?

The fact that a significant percentage of the common names of plants in the Brazilian northeast region exhibits correspondences to multiple species is irrevocably established. A more thorough understanding of the dynamics and dimensions of such semantic-biological variability and the corresponding implications requires the integration of several areas of knowledge, including taxonomy, biochemistry, population ecology, phytosociology, linguistics, and anthropology. 

The proportion of species found by ethnospecies (2.78) was significant, although we recognize that a more comprehensive coverage of markets and fairs in the nine northeastern states could lead to an increase of this proportion or even the emergence of new ethnospecies not listed in this survey. To what concerns the low number of respondents committed to the study, in all the six markets visited, it should be clarified that the purpose of the field survey was to catalogue ethnospecies currently marketed in order to support the identification of the corresponding species through the literature search.

## Figures and Tables

**Table 1:** Ethnospecies marketed in the Northeast Brazil and the corresponding species cataloged in the scientific literature.

Vernacular name	Family	Scientific name in the original source	Valid scientific name	Origin	Literature	State
Aroeira	Anacardiaceae	*Myracrodruon urundeuva* Allemão	*Myracrodruon urundeuva* Allemão	N	[1, 3, 9, 52–78]	PE, PB, SE, CE, PI, MA, RN, BA
		*Schinus terebinthifolius *Raddi	*Schinus terebinthifolius *Raddi	N	[1, 11, 65, 79–84]	PE, RN, BA

Cajá	Anacardiaceae	*Spondias mombin* L. *Spondias lutea* L.	*Spondias mombin* L.	N	[56, 57, 63, 67, 76, 82, 85–87]	PE, PB, PI, BA

Abre Caminho	Fabaceae	*Centrosema brasilianum * (L.) Benth.	*Centrosema brasilianum * (L.) Benth.	N	[88]	PB
	Schizaeaceae	*Lygodium venustum* Sw.	*Lygodium venustum* Sw.	N	[1, 65]	PE
		*Lygodium volubile* Sw.	*Lygodium volubile* Sw.	N	[1, 65]	PE

Açoita cavalo	Tiliaceae	*Luehea divaricata* Mart.	*Luehea divaricata *Mart.	N	[66, 68]	MA
		*Luehea ochrophylla* Mart.	*Luehea ochrophylla* Mart.	N	[89]	PB
		*Luehea grandiflora *Mart.	*Luehea grandiflora* Mart.	N	[61, 68]	MA
		*Luehea speciosa * Willd.				
		*Luehea candicans *Mart.	*Luehea candicans* Mart.	N	[57]	PI

Acônito	Amaranthaceae	*Pfaffia glomerata * (Spreng.) Pedersen	*Pfaffia glomerata * (Spreng.) Pedersen	N	[56, 65]	PE
		*Alternanthera brasiliana * (L.) Kuntze	*Alternanthera brasiliana * (L.) Kuntze	N	[77]	PB

Amburana	Fabaceae	*Amburana cearensis * (Allemão) A. C. Sm.	*Amburana cearensis * (Allemão) A. C. Sm.	N	[68–70, 78, 79]	PB, CE, MA, BA
	Burseraceae	*Commiphora leptophloeos * (Mart.) J. B. Gillett	*Commiphora leptophloeos * (Mart.) J. B. Gillett	N	[1, 3, 62–64, 70, 71, 73, 76, 80, 88]	PE, PB, RN, BA
		*Bursera leptophloeos *Mart.				

Cumaru	Fabaceae	*Amburana cearensis * (Allemão) A. C. Sm.	*Amburana cearensis * (Allemão) A. C. Sm.	N	[3, 52, 53, 55, 59, 60, 62, 67, 70–72, 76, 77]	PE, PB, CE, RN
		*Torresea cearensis *Allemão				
		*Dipteryx odorata * (Aubl.) Willd.	*Dipteryx odorata * (Aubl.) Willd.	N	[81]	RN

Angelica	Rubiaceae	*Guettarda platypoda *DC.	*Guettarda platypoda *DC.	N	[56, 89]	PE, PB
		*Guettarda angelica *Mart. ex Mull. Arg.	*Guettarda angelica *Mart. ex Mull. Arg.	N	[90]	RN

Araticum	Annonaceae	*Annona crassiflora *Mart.	*Annona crassiflora *Mart.	N	[85]	PB
		*Annona coriacea *Mart.	*Annona coriacea *Mart.	N	[78, 91]	PB, CE

Angico	Fabaceae	*Anadenanthera colubrina * (Vell.) Brenan	*Anadenanthera colubrina * (Vell.) Brenan	N	[3, 11, 52–54, 56, 59, 60, 62–64, 67, 69, 70, 72–74, 76–80, 85, 88, 92–94]	PE, PB, CE, PI, RN, BA
		*Anadenanthera macrocarpa * (Vell.) Brenan				
		*Piptadenia colubrina * (Vell.) Benth.				

Assa-peixe	Asteraceae	*Vernonia polyanthes * (Spreng.) Less.	*Vernonanthura phosphorica * (Vell.) H. Rob.	N	[79]	BA
		*Vernonia scabra *Pers.	*Vernonanthura brasiliana * (L.) H. Rob.	N	[60]	CE
		*Vernonia ferruginea *Less.	*Vernonanthura ferruginea * (Less.) H. Rob.	N	[82]	BA
		*Gochnatia velutina * (Bong.) Cabrera	*Gochnatia velutina * (Bong.) Cabrera	N	[79]	BA
		*Verbesina macrophylla *(Mull.) S. F. Blake	*Verbesina macrophylla *(Mull.) S. F. Blake	N	[83]	BA
	Euphorbiaceae	*Acalypha multicaulis *Mull. Arg.	*Acalypha multicaulis *Mull. Arg.	N	[95]	SE

Balaio de veio	Asteraceae	*Conocliniopsis prasiifolia * (DC.) R. M. King and H. Rob.	*Conocliniopsis prasiifolia * (DC.) R. M. King and H. Rob.	N	[58, 95, 96]	SE
		*Lourteigia ballotifolia * (Kunth) R. M. King and H. Rob.				
		*Centratherum punctatum* Cass.	*Centratherum punctatum* Cass.	N	[82]	BA
		*Ageratum conyzoides* L.	*Ageratum conyzoides* L.	N	[96]	SE
	Chrysobalanaceae	*Hirtella ciliata *Mart. and Zucc.	*Hirtella ciliata *Mart. and Zucc.	N	[78]	CE

Batata de Purga	Convolvulaceae	*Operculina alata *Urb.	*Operculina alata *Urb.	N	[11, 56, 66, 92]	PE, CE, MA
		*Operculina convolvulus *Silva Manso	*Operculina macrocarpa * (L.) Urb.	N	[9, 11, 57–59, 66, 94, 97]	PE, PB, SE, CE, PI, RN
		*Operculina macrocarpa * (L.) * Urb. *				
		*Ipomoea purga * (Wender.) Hayne	*Ipomoea dumosa * (Benth.) L. O. Williams	E	[68]	MA
		*Operculina hamiltonii * (G. Don) D. F. Austin and Staples	*Operculina hamiltonii * (G. Don) D. F. Austin and Staples	N	[72, 88]	PB

Burdão de velho	Fabaceae	*Pithecellobium saman * (Jacq.) Benth.	*Samanea saman * (Jacq.) Merr.	E	[56, 61]	PE, MA
		*Samanea tubulosa * (Benth.) Barneby and J. W. Grimes	*Samanea tubulosa * (Benth.) Barneby and J. W. Grimes	N	[86]	PB
		*Albizia polycephala * (Benth.) Killip	*Albizia polycephala * (Benth.) Killip	N	[85]	PB

Canafístula	Fabaceae	*Senna spectabilis * (DC.) H. S. Irwin and Barneby	*Senna spectabilis * (DC.) H. S. Irwin and Barneby	N	[3, 60, 67, 70, 75, 77, 90]	PE, PB, CE, RN
		*Senna martiana * (Benth.) H. S. Irwin and Barneby	*Senna martiana * (Benth.) H. S. Irwin and Barneby	N	[75]	PE
		*Albizia inundata * (Mart.) Barneby and J. W. Grimes	*Albizia inundata * (Mart.) Barneby and J. W. Grimes	N	[73]	PE
		*Peltophorum dubium * (Spreng.) Taub.	*Peltophorum dubium * (Spreng.) Taub.	N	[82]	BA

Capeba	Begoniaceae	*Begonia vitifolia *Schott	*Begonia reniformis *Dryand.	N	[1, 11, 56, 65, 94]	PE
		*Begonia reniformis *Dryand.				
		*Begonia huberi C.* DC.				
	Piperaceae	*Pothomorphe peltata * (L.) * Miq. *	*Piper peltatum* L.	N	[67]	BA
		*Piper marginatum *Jacq.	*Piper marginatum *Jacq.	N	[53]	PE
		*Piper umbellatum* L.	*Piper umbellatum* L.	N	[84]	BA

Murici	Malpighiaceae	*Byrsonima sericea *DC.	*Byrsonima sericea *DC.	N	[11, 56, 63, 82, 86, 87, 89, 98]	PE, PB, CE, PI, BA
		*Byrsonima verbascifolia * (L.) DC.	*Byrsonima verbascifolia * (L.) DC.	N	[98]	CE
		*Byrsonima coccolobifolia *Kunth	*Byrsonima coccolobifolia *Kunth	N	[98]	CE
		*Byrsonima gardneriana* A. Juss.	*Byrsonima gardneriana* A. Juss.	N	[74, 85]	PB, PI
		*Byrsonima correifolia* A. Juss.	*Byrsonima correifolia* A. Juss.	N	[57]	PI

Mulungu	Fabaceae	*Erythrina velutina *Willd.	*Erythrina velutina *Willd.	N	[1, 3, 56, 59, 60, 63, 67, 71–73, 75–77, 86, 88, 89, 93, 94, 97]	PE, PB, SE, CE, RN

Muçambê	Cleomaceae	*Cleome hassleriana *Chodat	*Tarenaya hassleriana * (Chodat) Iltis	N	[94]	PE
		*Cleome spinosa *Jacq.	*Tarenaya spinosa * (Jacq.) Raf.	N	[3, 9, 56, 57, 59, 65, 71, 72, 75, 92, 93]	PE, PB, CE, PI, RN

Mororó	Fabaceae	*Bauhinia cheilantha * (Bong.) Steud.	*Bauhinia cheilantha * (Bong.) Steud.	N	[3, 58, 60, 62–64, 71–73, 75–78, 85, 90, 95, 99]	PE, PB, SE, CE, RN
		*Bauhinia forficata *Link	*Bauhinia forficata *Link	N	[57, 68, 81, 93]	PE, MA, RN
		*Bauhinia subclavata *Benth.	*Bauhinia subclavata *Benth.	N	[80]	BA
		*Bauhinia smilacifolia Burch. *ex Benth.	*Bauhinia smilacifolia Burch. *ex Benth.	N	[90]	RN
		*Bauhinia outimouta *Aubl.	*Phanera outimouta * (Aubl.) L. P. Queiroz	N	[78]	CE
		*Bauhinia acuruana *Moric.	*Bauhinia acuruana *Moric.	N	[74]	PI
		*Bauhinia ungulata* L.	*Bauhinia ungulata* L.	N	[55]	CE

Piqui	Caryocaraceae	*Caryocar brasiliense *Cambess.	*Caryocar brasiliense *Cambess.	N	[61, 66, 68]	MA
		*Caryocar coriaceum *Wittm.	*Caryocar coriaceum *Wittm.	N	[78, 92, 98]	CE

Quebra pedra	Euphorbiaceae	*Phyllanthus niruri* L.	*Phyllanthus niruri* L.	N	[1, 11, 57, 59, 61, 62, 65, 66, 68, 71, 75, 84, 90, 99]	PE, PB, PI, MA, RN, BA
		*Phyllanthus amarus *Schumach. and Thonn.	*Phyllanthus amarus *Schumach. and Thonn.	N	[9, 52, 53, 55, 56, 63, 72, 76, 92–94]	PE, PB, CE
		*Phyllanthus tenellus *Roxb.	*Phyllanthus tenellus* Roxb.	N	[79, 83]	BA
		*Phyllanthus corcovadensis *Mull. Arg.				
		*Euphorbia hyssopifolia* L.	*Euphorbia hyssopifolia* L.	N	[75, 95]	PE, SE
		Cham*aesyce hyssopifolia * (L.) Small				
		*Euphorbia thymifolia* L.	*Euphorbia thymifolia* L.	N	[56]	PE
		*Euphorbia prostrata *Aiton	*Euphorbia prostrata *Aiton	N	[75]	PE
		*Phyllanthus flaviflorus * (K. Schum. and Lauterb.) Airy Shaw	*Phyllanthus flaviflorus * (K. Schum. and Lauterb.) Airy Shaw	E	[69]	BA
		*Phyllanthus urinaria* L.	*Phyllanthus urinaria* L.	E	[78]	CE
	Oxalidaceae	*Oxalis divaricata *Mart. ex Zucc.	*Oxalis divaricata *Mart. ex Zucc.	N	[58]	SE

Sabugueiro	Adoxaceae	*Sambucus australis *Cham. and Schltdl.	*Sambucus australis *Cham. and Schltdl.	N	[9, 11, 56, 78, 81, 83, 84, 93, 94, 100]	PE, PB, CE, RN, BA
		*Sambucus racemosa* L.	*Sambucus racemosa* L.	E	[69]	BA
		*Sambucus nigra* L.	*Sambucus nigra* L.	E	[1, 11, 65, 67, 76, 79, 92, 99]	PE, CE, RN, BA

Fedegoso	Boraginaceae	*Heliotropium indicum* L.	*Heliotropium indicum* L.	N	[1, 11, 52, 55, 56, 62, 63, 71, 75, 76, 85, 88–90, 92, 94]	PE, PB, CE, RN
		*Heliotropium elongatum *Hoffm. ex Roem. and Schult.	*Heliotropium elongatum *Hoffm. ex Roem. and Schult.	N	[3, 53, 59, 72]	PE, PB, RN
		*Heliotropium procumbens *Mill.	*Heliotropium procumbens *Mill.	E	[60]	CE
	Fabaceae	*Senna occidentalis * (L.) Link	*Senna occidentalis * (L.) Link	N	[57, 67, 69, 80, 83, 84, 88, 95]	PB, SE, PI, BA
		*Senna uniflora * (Mill.) H. S. Irwin and Barneby	*Senna uniflora * (Mill.) H. S. Irwin and Barneby	N	[66]	MA

Favela	Euphorbiaceae	*Cnidoscolus quercifolius *Pohl	*Cnidoscolus quercifolius *Pohl	N	[3, 9, 58–60, 62, 70, 71, 74, 77, 88]	PB, SE, CE, PI, RN
		*Cnidoscolus phyllacanthus * (Mull. Arg.) Pax and L. Hoffm.			[9, 60, 71, 74, 88]	PB, CE, PI

Velame	Euphorbiaceae	*Croton rhamnifolius *Willd.	*Croton heliotropiifolius *Kunth	N	[3, 57, 59, 62–64, 70, 73, 75, 76, 80, 85, 94, 95]	PE, PB, SE, PI, RN, BA
		*Croton heliotropiifolius *Kunth				
		*Croton moritibensis *Baill.				
		*Croton sonderianus *Mull. Arg.	*Croton sonderianus *Mull. Arg.	N	[61, 93]	PE, MA
		*Croton campestris *A. St.-Hil.	*Croton campestris *A. St.-Hil.	N	[69, 71, 74, 78, 92]	PB, CE, PI, BA
		*Croton tenuifolius *Pax and K. Hoffm.	*Croton betaceus *Baill.	N	[57]	PI

Acansu	Fabaceae	*Periandra dulcis *Mart. ex Benth.	*Periandra mediterranea * (Vell.) Taub.	N	[53, 80, 89]	PE, PB, BA
		*Periandra mediterranea * (Vell.) Taub.				

Chanana	Turneraceae	*Turnera ulmifolia* L.	*Turnera ulmifolia* L.	E	[1, 9, 11, 56, 57, 60, 61, 65, 66, 68, 71, 89]	PE, PB, CE, PI, MA
		*Turnera subulata *Sm.	*Turnera subulata *Sm.	N	[55, 59, 62, 78, 88, 89, 95]	PB, SE, CE, RN
		*Turnera chamaedrifolia *Cambess.	*Turnera chamaedrifolia *Cambess.	N	[77]	PB
		*Turnera guianensis * Aubl.	*Turnera guianensis *Aubl.	N	[68]	MA

João Mole	Nyctaginaceae	*Guapira opposita * (Vell.) Reitz	*Guapira opposita * (Vell.) Reitz	N	[85, 86]	PB
		*Guapira noxia * (*Netto*) Lundell	*Guapira noxia * (*Netto*) Lundell	N	[95]	SE

Unha de gato	Lycopodiaceae	*Lycopodiella cernua * (L.) Pic. Serm.	*Lycopodiella cernua * (L.) * Pic. Serm. *	N	[56]	PE
	Alismataceae	*Echinochloa colona * (L.) Link	*Echinochloa colona * (L.) Link	E	[75]	PE
	Rubiaceae	*Uncaria tomentosa * (Willd.) DC.	*Uncaria tomentosa * (Willd.) DC.	N	[11, 54, 68, 72]	PE, PB, MA
	Fabaceae	*Acacia paniculata *Willd.	*Senegalia tenuifolia * (L.) * Britton *and* Rose *	N	[9, 52, 60, 63, 64, 73, 76]	PE, CE
		*Mimosa somnians Humb. *and Bonpl. ex Willd.	*Mimosa somnians Humb. *and Bonpl. ex Willd.	N	[95]	SE
		*Mimosa sensitiva* L.	*Mimosa sensitiva* L.	N	[58]	SE

Lingua de Vaca	Asteraceae	*Elephantopus mollis *Kunth	*Elephantopus mollis *Kunth	E	[66]	MA
	Portulacaceae	*Talinum portulacifolium * (Forssk.) * Asch. *ex* Schweinf. *	*Talinum portulacifolium * (Forssk.) * Asch. *ex* Schweinf. *	E	[58]	SE
	Fabaceae	*Centrosema brasilianum * (L.) Benth.	*Centrosema brasilianum * (L.) Benth.	N	[90]	RN
	Sapotaceae	*Chrysophyllum splendens *Spreng.	*Chrysophyllum splendens *Spreng.	N	[82]	BA
	Alismataceae	*Echinodorus subalatus * (Mart.) Griseb.	*Echinodorus subalatus * (Mart.) Griseb.	N	[59]	RN

Lacre	Clusiaceae	*Vismia guianensis * (Aubl.) Pers.	*Vismia guianensis * (Aubl.) Pers.	N	[1, 11, 53, 56, 65, 78, 86, 89, 94]	PE, PB, CE
		*Vismia brasiliensis *Choisy	*Vismia brasiliensis *Choisy	N	[87]	PI

Jurubeba	Solanaceae	*Solanum paniculatum* L.	*Solanum paniculatum* L.	N	[1, 11, 52, 53, 56, 63, 67, 74–77, 80, 86, 88, 93, 94]	PE, PB, PI, BA
		*Solanum paludosum *Moric.	*Solanum paludosum *Moric.	N	[58, 89, 90]	PB, SE, RN
		*Solanum absconditum *Agra	*Solanum absconditum* Agra	N	[85]	PB
		*Solanum auriculatum* Aiton	*Solanum mauritianum* Scop.	N	[97]	SE
		*Solanum erianthum * D. Don	*Solanum granuloso-leprosum* Dunal	N	[78]	CE
		*Solanum polytrichum* Moric.	*Solanum polyrtrichum* Moric.	N	[82]	BA
		*Solanum albidum *Dunal	*Solanum albidum *Dunal	E	[55]	CE
		*Solanum tabacifolium *Vell.	*Solanum scuticum *M. Nee	N	[79]	BA
		*Solanum lycocarpum *A. St.-Hil.	*Solanum lycocarpum *A. St.-Hil.	N	[66]	MA

Cedro	Meliaceae	*Cedrela fissilis *Vell.	*Cedrela fissilis *Vell.	N	[80, 86, 93]	PE, PB, BA
		*Cedrela odorata* L.	*Cedrela odorata* L.	N	[1, 11, 52, 53, 56, 63, 72, 73, 76, 84, 85]	PE, PB, BA
	Tiliaceae	*Luehea grandiflora *Mart.	*Luehea grandiflora *Mart.	N	[69]	BA

Crista de galo	Amaranthaceae	*Celosia cristata* L.	*Celosia argentea* L.	E	[61, 63, 94]	PE, MA
	Plumbaginaceae	*Plumbago scandens* L.	*Plumbago scandens* L.	N	[95]	SE
	Boraginaceae	*Heliotropium indicum* L.	*Heliotropium indicum* L.	N	[58, 78, 83, 88]	PB, SE, CE, BA
		*Heliotropium angiospermum *Murray	*Heliotropium angiospermum *Murray	N	[11]	PE
		*Heliotropium tiaridioides *Cham.	*Heliotropium transalpinum *Vell.	N	[74]	PI

Manjerona	Lamiaceae	*Ocimum americanum* L.	*Ocimum americanum* L.	E	[1, 56, 65]	PE
		*Origanum majorana* L.	*Origanum majorana* L.	E	[66, 84, 99, 100]	PB, MA, RN, BA

Angelim	Fabaceae	*Andira nitida *Mart. ex Benth.	*Andira nitida *Mart. ex Benth.	N	[56]	PE
		*Piptadenia obliqua * (Pers.) J. F. Macbr.	*Pityrocarpa obliqua subsp. brasiliensis * (G. P. Lewis) Luckow and R. W. Jobson	N	[60]	CE
		*Andira vermifuga *Mart. ex Benth.	*Andira vermifuga *Mart. ex Benth.	N	[74]	PI
		*Andira paniculata *Benth.	*Andira paniculata* Benth.	N	[87]	PI
		*Luetzelburgia auriculata * (Allemão) Ducke	*Luetzelburgia auriculata * (Allemão) Ducke	N	[87]	PI

Arrozinho	Polygalaceae	*Polygala gracilis *Kunth	*Polygala gracilis *Kunth	N	[88]	PB
		*Polygala paniculata* L.	*Polygala paniculata* L.	N	[88]	PB
	Fabaceae	*Zornia latifolia *Sm.	*Zornia latifolia Sm. *	N	[67]	BA

Anil estrelado	Schisandraceae	*Illicium verum *Hook. f.	*Illicium verum *Hook. f.	E	[1, 11, 53]	PE

Cavalinha	Equisetaceae	*Equisetum hyemale* L.	*Equisetum hyemale* L.	E	[54]	PB
		*Equisetum giganteum* L.	*Equisetum giganteum* L.	N	[93]	PE
		*Equisetum arvense* L.	*Equisetum arvense* L.	E	[84]	BA

Chumbinho	Verbenaceae	*Lantana camara* L.	*Lantana camara* L.	N	[11, 53, 56, 58, 63, 64, 67, 73, 76, 82, 86, 88–90, 95, 96, 98]	PE, PB, SE, CE, RN, BA
	Oxalidaceae	*Oxalis insipida A. *St.-Hill.	*Oxalis psoraleoides *Kunth	N	[73]	PE
	Sapindaceae	*Cardiospermum corindum* L.	*Cardiospermum corindum* L.	N	[74]	PI
		*Cardiospermum halicacabum* L.	*Cardiospermum halicacabum* L.	N	[74]	PI

Camará	Verbenaceae	*Lantana camara* L.	*Lantana camara* L.	N	[60, 67, 71, 74, 88]	PB, CE, PI, BA
		*Lantana canescens *Kunth	*Lantana canescens *Kunth	N	[58]	SE
	Asteraceae	*Wedelia scaberrima *Benth.	Wedelia calycina Rich.	N	[90]	RN
		*Verbesina diversifolia* DC.	*Verbesina diversifolia* DC.	N	[86]	PB

Canela de velho	Melastomataceae	*Miconia albicans * (Sw.) Steud.	*Miconia albicans * (Sw.) Steud.	N	[67]	BA
	Fabaceae	*Cenostigma *Gardner*ianum *Tul.	*Cenostigma macrophyllum *Tul.	N	[74]	PI
	Primulaceae	*Cybianthus detergens *Mart.	*Cybianthus detergens *Mart.	N	[78]	CE

Catuaba	Bignoniaceae	*Anemopaegma arvense * (Vell.) Stellfeld and J. F. Souza	*Anemopaegma arvense * (Vell.) Stellfeld and J. F. Souza	N	[68]	MA
	Erythroxylaceae	*Erythroxylum amplifolium * (Mart.) O. E. Schulz	*Erythroxylum amplifolium * (Mart.) O. E. Schulz	N	[78]	CE
		* Erythroxylum vacciniifolium *Mart.	* Erythroxylum vacciniifolium *Mart.	N	[66, 69]	MA, BA

Japecanga	Smilacaceae	*Smilax campestris Griseb. *	*Smilax campestris Griseb. *	N	[78]	CE
		*Smilax japecanga Griseb. *	*Smilax japecanga Griseb. *	N	[98]	CE
		*Smilax cissoides *Mart. ex* Griseb. *	*Smilax cissoides *Mart. ex* Griseb. *	N	[85]	PB
		*Smilax rotundifolia* L.	*Smilax rotundifolia* L.	N	[11]	PE

Vassourinha	Plantaginaceae	*Scoparia dulcis* L.	*Scoparia dulcis* L.	N	[9, 59, 61, 66, 67, 71, 74, 78, 80, 83, 88]	PB, CE, PI, MA, RN, BA
	Asteraceae	*Emilia sonchifolia * (L.) DC.	*Emilia sonchifolia * (L.) DC.	N	[93]	PE
	Brassicaceae	*Nasturtium officinale* W. T. Aiton	*Nasturtium officinale* W. T. Aiton	E	[93]	PE
	Scrophulariaceae	*Capraria biflora* L.	*Capraria biflora* L.	N	[60]	CE
	Polygalaceae	*Polygala paniculata* L.	*Polygala paniculata* L.	N	[67]	BA
	Rubiaceae	*Borreria scabiosoides *Cham. and Schldl.	*Borreria scabiosoides *Cham. and Schldl.	N	[89]	PB
		*Spermacoce verticillata* L.	*Borreria verticillata * (L.) * G. Mey. *	N	[57]	PI

Transagem	Plantaginaceae	*Plantago major* L.	*Plantago major* L.	E	[53, 54, 67, 69, 72, 79, 83, 84, 94]	PE, PB, BA
	Alismataceae	*Echinodorus grandiflorus * (Cham. and* Schltd* L.) Micheli	*Echinodorus grandiflorus * (Cham. and* Schltd* L.) Micheli	N	[76, 93]	PE

Alcachofra	Asteraceae	*Vernonia condensata *Baker	*Gymnanthemum amygdalinum * (Delile) Sch. Bip. ex Walp.	N	[1, 53, 56, 63, 67, 76, 94]	PE
		*Cynara scolymus* L.	*Cynara cardunculus* L.	E	[52, 63, 84]	PE, BA
		*Gymnanthemum amygdalinum * (Delile) Sch. Bip. ex Walp.	*Gymnanthemum amygdalinum * (Delile) Sch. Bip. ex Walp.	N	[11]	PE

Açafrão	Zingiberaceae	*Curcuma longa* L.	*Curcuma longa* L.	E	[72, 84]	PB, BA

Alfavaca	Lamiaceae	*Ocimum basilicum* L.	*Ocimum basilicum* L.	E	[68, 81]	MA, RN
		*Ocimum campechianum *Mill.	*Ocimum campechianum *Mill.	N	[9, 53, 60, 83]	PE, CE, BA
		*Ocimum gratissimum* L.	*Ocimum gratissimum* L.	E	[1, 55, 56, 61, 78]	PE, CE, MA

Catolé	Arecaceae	*Syagrus oleracea * (Mart.) * Becc. *	*Syagrus oleracea * (Mart.) Becc.	N	[85]	PB
		*Syagrus picrophylla *Barb. Rodr.	*Syagrus picrophylla *Barb. Rodr.	N	[55]	CE
		*Syagrus cearensis *Noblick	*Syagrus cearensis *Noblick	N	[78]	CE

Mentrasto	Asteraceae	*Ageratum conyzoides* L.	*Ageratum conyzoides* L.	N	[58–60, 69, 78, 83, 94, 99]	PE, SE, CE, RN, BA
		*Stilpnopappus scaposus *DC.	*Stilpnopappus scaposus *DC.	N	[96]	SE
		*Blainvillea rhomboidea *Cass.	*Blainvillea acmella * (L.) Philipson	N	[96]	SE
		*Prolobus nitidulus * (Baker) R. M. King and H. Rob.	*Prolobus nitidulus * (Baker) R. M. King and H. Rob.	N	[96]	SE
	Polygalaceae	*Polygala violacea *Aubl.	*Polygala violacea *Aubl.	N	[95]	SE

Catingueira	Fabaceae	*Caesalpinia pyramidalis Tul*.	*Poincianella pyramidalis * (Tul.) L. P. Queiroz	N	[3, 9, 11, 52, 53, 56, 58, 59, 62, 63, 67, 70, 71, 75–77, 88, 90, 95, 99]	PE, PB, SE, CE, RN
		*Poincianella pyramidalis * (Tul.) L. P. Queiroz				
		*Caesalpinia bracteosa *Tul.	*Poincianella bracteosa * (Tul.) L. P. Queiroz	N	[57, 60]	CE, PI
		*Poincianella microphylla * (Mart. ex G. Don) L. P. Queiroz	*Poincianella microphylla * (Mart. ex G. Don) L. P. Queiroz	N	[80]	BA

Marmeleiro	Euphorbiaceae	*Croton blanchetianus Baill*.	*Croton blanchetianus *Baill.	N	[3, 52, 59, 63, 64, 70, 73, 76, 78, 80, 95]	PE, PB, SE, CE, RN, BA
		*Croton sonderianus *Mull. Arg.	*Croton sonderianus *Mull. Arg.	N	[55, 62, 71, 72, 74, 75, 81, 90]	PE, PB, CE, PI, RN
		*Croton rhamnifolius *Willd.	*Croton heliotropiifolius *Kunth	N	[98]	CE
		*Croton urticifolius *Lam.	*Croton urticifolius *Lam.	N	[86]	PB
		*Croton argyrophylloides *Mull. Arg.	*Croton argyrophylloides *Mull. Arg.	N	[76]	PE

Oiticica	Chrysobalanaceae	*Licania rigida *Benth.	*Licania rigida *Benth.	N	[9, 55, 59, 60, 70–72, 77, 85, 99]	PB, CE, RN

Picão	Asteraceae	*Bidens pilosa* L.	*Bidens pilosa* L.	E	[61, 67]	MA, BA

Barriguda	Malvaceae	*Ceiba glaziovii * (*Kuntze*) K. Schum.	*Ceiba glaziovii * (Kuntze) K. Schum.	N	[9, 77, 85, 88]	PB, CE
	Bombacaceae	*Chorisia glaziovii * (Kuntze) E. Santos	*Chorisia glaziovii * (Kuntze) E. Santos	N	[63, 64, 73, 75]	PE
	Lamiaceae	*Hypenia salzmannii * (Benth.) Harley	*Hypenia salzmannii * (Benth.) Harley	N	[57]	PI

Vique	Polygalaceae	*Polygala paniculata* L.	*Polygala paniculata* L.	N	[67, 90]	RN, BA
		*Polygala bryoides *A. St.-Hil. and Moq.	*Polygala bryoides *A.St.-Hil. and Moq.	N	[90]	RN
	Lamiaceae	*Mentha spicata* L.	*Mentha spicata* L.	E	[66, 68]	MA
		*Mentha pulegium* L.	*Mentha pulegium* L.	E	[56]	PE

Agrião	Brassicaceae	*Nasturtium officinale *W. T. Aiton	*Nasturtium officinale *W. T. Aiton	E	[9, 53, 56, 69, 81, 93, 94, 99]	PE, CE, RN, BA
		*Rorippa pumila* (Camb.) A. Lima	*Rorippa pumila* (Camb.) A. Lima	E	[65]	PE
	Asteraceae	*Acmella ciliata * (Kunth) Cass.	*Acmella ciliata * (Kunth) Cass.	N	[57]	PI
		*Acmella oleracea * (L.) R. K. Jansen	*Acmella oleracea * (L.) R. K. Jansen	N	[72]	PB

Algodão	Malvaceae	*Gossypium hirsutum* L.	*Gossypium hirsutum* L.	E	[57, 78, 93]	PE, CE, PI
		*Gossypium barbadense* L.	*Gossypium barbadense* L.	E	[56, 67, 78]	PE, CE, BA
		*Gossypium herbaceum* L.	*Gossypium herbaceum* L.	E	[66, 68, 69, 75, 83, 84]	PE, MA, BA
		*Gossypium arboreum* L.	*Gossypium arboreum* L.	E	[61]	MA

Ameixa	Olacaceae	*Ximenia americana* L.	*Ximenia americana* L.	N	[3, 9, 11, 53, 55, 57–60, 62, 70, 74, 78, 90, 97]	PE, PB, SE, CE, PI, RN
	Sapotaceae	*Chrysophyllum arenarium *Allemão	*Chrysophyllum arenarium *Allemão	N	[98]	CE
	Myrtaceae	*Eugenia cumini * (L.) Druce	*Syzygium cumini * (L.) Skeels	E	[68]	MA
	Rosaceae	*Prunus domestica* L.	*Prunus domestica* L.	E	[81]	RN

Anador	Lamiaceae	*Plectranthus barbatus *Andrews	*Plectranthus barbatus *Andrews	E	[68]	MA
		*Ocimum selloi *Benth.	*Ocimum carnosum * (Spreng.) Link and* Otto *ex Benth.	N	[67]	BA
	Acanthaceae	*Justicia gendarussa * Burm. f.	*Justicia gendarussa * Burm. f.	E	[53]	PE
		*Justicia pectoralis *Jacq.	*Justicia pectoralis *Jacq.	N	[52, 55, 63, 94]	PE, CE
	Amaranthaceae	*Alternanthera brasiliana * (L.) Kuntze	*Alternanthera brasiliana * (L.) Kuntze	N	[67, 69]	BA
		*Pfaffia glomerata * (Spreng.) Pedersen	*Pfaffia glomerata * (Spreng.) Pedersen	N	[67, 79]	BA
	Asteraceae	*Artemisia vulgaris* L.	*Artemisia vulgaris* L.	E	[72, 78]	PB, CE
		*Iodina rhombifolia *Hook. and Arn.	*Jodina rhombifolia * (Hook. and Arn) Reissek	N	[100]	PB

Arruda	Rutaceae	*Ruta graveolens* L.	*Ruta graveolens* L.	E	[1, 9, 11, 52–57, 63, 65, 67–69, 72, 75, 76, 78, 79, 81, 83, 84, 93, 94, 99–101]	PE, PB, SE, CE, PI, MA, RN, BA

Artemísia	Asteraceae	*Artemisia vulgaris* L.	*Artemisia vulgaris* L.	E	[54, 69, 83, 84, 94]	PE, PB, BA

Bambu	Poaceae	*Dendrocalamus giganteus *Wall. ex* Munro *	*Dendrocalamus giganteus *Wall. ex* Munro *	E	[11, 56]	PE
		*Bambusa arundinacea * (Retz.) Willd.	*Bambusa bambos * (L.) Voss	E	[69]	BA
		*Bambusa vulgaris* Schrad. ex J. C. Wendl.	*Bambusa vulgaris* Schrad. ex J. C. Wendl.	E	[84]	BA

Janauba	Apocynacaee	*Himatanthus bracteatus * (*A. *DC.) Woodson	*Himatanthus bracteatus * (*A. *DC.) Woodson	N	[82]	BA
		*Himatanthus sucuuba * (Spruce ex Mull. Arg.) Woodson	*Himatanthus sucuuba * (Spruce ex Mull. Arg.) Woodson	N	[66]	MA
		*Himatanthus drasticus *(Mart.) Plumel	*Himatanthus drasticus* (Mart.) Plumel	N	[78, 98]	CE

Barbatimão	Fabaceae	*Stryphnodendron adstringens* (Mart.) Coville	*Stryphnodendron adstringens* (Mart.) Coville	N	[1, 79, 92, 94, 99]	PE, CE, RN, BA
		*Stryphnodendron barbatimam *Mart.				
		*Abarema cochliacarpos * (Gomes) Barneby and J. W.	*Abarema cochliacarpos * (Gomes) Barneby and J. W.	N	[11, 53, 56, 69]	PE, BA
		*Pithecellobium cochliacarpum * (Gomes) J. F. Macbr.				
		*Stryphnodendron coriaceum *Benth.	*Stryphnodendron coriaceum *Benth.	N	[54, 71, 78, 87, 98]	PB, CE, PI

Bom nome	Celastraceae	*Maytenus rigida *Mart.	*Maytenus rigida *Mart.	N	[1, 3, 9, 52–54, 56, 58, 60, 63, 67, 71, 73, 75–77, 88, 95–97]	PE, PB, SE, CE
		*Maytenus distichophylla *Mart.	*Maytenus distichophylla *Mart.	N	[78]	CE

Caju	Anacardiaceae	*Anacardium occidentale* L.	*Anacardium occidentale* L.	N	[9, 11, 54, 56–58, 61, 63, 66–68, 71, 75, 78, 79, 81–84, 86, 87, 89, 92–94, 99]	PE, PB, SE, CE, PI, MA, RN, BA

Cardo santo	Papaveraceae	*Argemone mexicana* L.	*Argemone subfusiformis *G. B. Ownbey	E	[1, 11, 53, 60, 71, 75, 77, 79, 83, 88, 93]	PE, PB, CE, BA
		*Argemone subfusiformis *G. B. Ownbey				
	Asteraceae	*Carduus benedictus *Gaert.	*Carduus benedictus *Gaert.	E	[84]	BA

Candeia	Fabaceae	*Plathymenia reticulata *Benth.	*Plathymenia reticulata *Benth.	N	[61, 74]	PI, MA
	Asteraceae	*Gochnatia oligocephala * (Gardner) Cabrera	*Gochnatia oligocephala * (Gardner) Cabrera	N	[80]	BA

Canela	Lamiaceae	*Cinnamomum zeylanicum *Blume	*Cinnamomum verum* J. Presl	E	[11, 53, 63, 69, 76, 81, 83, 84, 93]	PE, RN, BA
	Lauraceae	*Nectandra cuspidata *Nees and Mart.	*Nectandra cuspidata *Nees and Mart.	N	[56]	PE
		*Nectandra leucantha *Nees and Mart.	*Nectandra leucantha *Nees and Mart.	N	[94]	PE

Mandacaru	Cactaceae	*Cereus jamacaru *DC.	*Cereus jamacaru *DC.	N	[1, 9, 52, 53, 56, 58–60, 63, 71, 75, 76, 78, 80, 88, 93–95]	PE, PB, SE, CE, RN, BA
		*Opuntia ficus-indica * (L.) Mill.	*Opuntia ficus-indica * (L.) Mill.	E	[66]	MA

Carqueja	Asteraceae	*Baccharis trimera * (Less.) DC.	*Baccharis crispa *Spreng.	N	[1, 68, 79, 83, 99]	PE, MA, RN, BA

Cidreira	Verbenaceae	*Lippia alba * (Mill.) N. E. Br. ex Britton and P. Wilson	*Lippia alba * (Mill.) N. E. Br. ex Britton and P. Wilson	N	[1, 3, 9, 11, 52, 53, 55, 57, 58, 61–63, 67–69, 72, 75, 76, 78, 79, 83, 93–95, 99, 100]	PE, PB, SE, CE, PI, MA, RN, BA
		*Lippia citriodora *Kunth	*Aloysia citriodora *Palau	E	[97]	SE
	Lamiaceae	*Melissa officinalis* L.	*Melissa officinalis* L.	E	[66, 81, 84, 92, 93]	PE, CE, MA, RN, BA

Pra tudo	Crassulaceae	*Kalanchoe brasiliensis *Cambess.	*Kalanchoe crenata * (Andrews) Haw.	E	[75]	PE
	Sapindaceae	*Cardiospermum halicacabum* L.	*Cardiospermum halicacabum* L.	N	[77]	PB
	Rutaceae	*Zanthoxylum hamadryadicum* Pirani	*Zanthoxylum hamadryadicum* Pirani	N	[74]	PI
	Fabaceae	*Acosmium dasycarpum * (Vogel) * Yakovlev *	*Leptolobium dasycarpum *Vogel	N	[78]	CE

Copaiba	Fabaceae	*Copaifera langsdorffii* Desf.	*Copaifera langsdorffii* Desf.	N	[61, 66]	MA
		*Copaifera coriacea *Mart.	*Copaifera coriacea *Mart.	N	[87]	PI
		*Copaifera reticulata *Ducke	*Copaifera reticulata *Ducke	N	[68]	MA
		*Copaifera officinalis* (Jacq.) * *L.	*Copaifera officinalis* (Jacq.) * *L.	N	[84]	BA
		*Copaifera lucens *Dwyer	*Copaifera lucens *Dwyer	N	[69]	BA

Courama	Malvaceae	*Malvaviscus arboreus* Cav.	*Malvaviscus arboreus* Cav.	E	[81]	RN
	Crassulaceae	*Kalanchoe brasiliensis *Cambess.	*Kalanchoe crenata * (Andrews) Haw.	E	[53, 55, 56, 65]	PE, CE
		*Kalanchoe blossfeldiana *Poelln.	*Kalanchoe blossfeldiana* Poelln.	E	[94]	PE
		*Bryophyllum pinnatum * (Lam.) Oken	*Kalanchoe pinnata * (Lam.) Pers.	E	[1, 65, 72, 84, 99]	PE, PB, RN, BA
		*Bryophyllum calycinum *Salisb.				
		*Kalanchoe pinnata * (Lam.) Pers.				

Cordão de São Francisco	Lamiaceae	*Leonotis nepetifolia * (L.) R. Br.	*Leonotis nepetifolia * (L.) R. Br.a	E	[9, 57, 60, 67, 68, 71]	PB, CE, PI, MA, BA
		*Leucas martinicensis * (Jacq.) R. Br.	*Leucas martinicensis * (Jacq.) R. Br.	E	[77]	PB

Embaúba	Urticaceae	*Cecropia palmata *Willd.	*Cecropia palmata *Willd.	N	[86]	PB
		*Cecropia pachystachya *Trécul	*Cecropia pachystachya *Trécul	N	[61, 67, 82, 85]	PB, MA, BA
		*Cecropia peltata* L.	*Cecropia peltata* L.	N	[74]	PI

Imbiriba	Annonaceae	*Guatteria australis *A. St.-Hil.	*Guatteria australis *A. St.-Hil.	N	[9]	CE
	Lecythidaceae	*Eschweilera ovata * (Cambess.) Miers	*Eschweilera ovata * (Cambess.) Miers	N	[56, 85, 86, 89]	PE, PB

Espinheira santa	Fabaceae	*Zollernia ilicifolia * (Brongn.) Vogel	*Zollernia ilicifolia * (Brongn.) Vogel	N	[53]	PE
	Celastraceae	*Maytenus ilicifolia *Mart. ex Reissek	*Maytenus ilicifolia *Mart. ex Reissek	N	[68, 72, 79]	PB, MA, BA

Gengibre	Zingiberaceae	*Zingiber officinale *Roscoe	*Zingiber officinale *Roscoe	E	[53, 57, 66, 68, 72, 84, 93, 94]	PE, PB, PI, MA, BA

Graviola	Annonaceae	*Annona muricata* L.	*Annona muricata* L.	E	[3, 9, 11, 56, 63, 69, 75, 83, 93, 94, 99]	PE, PB, CE, RN, BA
		*Rollinia sericea * (R. E. Fr.) R. E. Fr.	*Annona neosericea *H. Rainer	N	[67]	BA
		*Annona cherimola * Mill.	*Annona cherimola * Mill.	E	[84]	BA

Jaboticaba	Myrtaceae	*Myrciaria cauliflora * (Mart.) O. Berg	*Plinia cauliflora * (Mart.) Kausel	N	[52, 63, 69, 76]	PE, BA

Juá	Rhamnaceae	*Ziziphus joazeiro *Mart.	*Ziziphus joazeiro *Mart.	N	[3, 9, 56, 57, 59, 60, 62–64, 67, 68, 70–72, 74–76, 78, 80, 81, 85, 86, 88, 89, 92, 93, 95]	PE, PB, SE, CE, PI, MA, RN, BA
		*Ziziphus cotinifolia *Reissek	*Ziziphus cotinifolia *Reissek	N	[77, 88]	PB

Louro	Lauraceae	*Laurus nobilis* L.	*Laurus nobilis* L.	E	[81, 84, 93]	PE, RN, BA
		*Ocotea glomerata * (Nees) Mez	*Ocotea glomerata * (Nees) Mez	N	[56]	PE
		*Ocimum gratisssimum* L.	*Ocimum gratisssimum* L.	E	[52, 63, 76, 94]	PE
		*Ocotea duckei* Vattimo	*Ocotea duckei *Vattimo	N	[89]	PB
		*Laurus azorica * (Seub.) Franco	*Laurus azorica * (Seub.) Franco	E	[100]	PB

Erva doce	Apiaceae	*Pimpinella anisum* L.	*Pimpinella anisum* L.	E	[1, 3, 11, 52, 56, 63, 68, 76, 78, 79, 81, 83, 92, 93, 100]	PE, PB, CE, MA, RN, BA
		*Foeniculum vulgare *Mill.	*Foeniculum vulgare *Mill.	E	[53, 67, 69, 84, 94]	PE, BA

Endro	Apiaceae	*Foeniculum vulgare *Mill.	*Foeniculum vulgare *Mill.	E	[1, 3, 11, 52, 63, 78]	PE, PB, CE
		*Anethum graveolens* L.	*Anethum graveolens* L.	E	[53, 54, 57, 72, 93, 100]	PE, PB, PI

Alecrim	Lamiaceae	*Rosmarinus officinalis* L.	*Rosmarinus officinalis* L.	E	[3, 11, 52, 53, 56, 63, 65, 68, 69, 72, 75, 76, 78, 79, 84, 92, 93, 99, 100]	PE, PB, CE, MA, RN, BA
	Fabaceae	*Calliandra depauperata *Benth.	*Calliandra depauperata *Benth.	N	[60]	CE
	Verbenaceae	*Lippia thymoides *Mart. and Schauer	*Lippia thymoides *Mart. and Schauer	N	[80]	BA

Abacate	Lauraceae	*Persea americana * Mill.	*Persea americana * Mill.	E	[11, 63, 66, 67, 67–69, 76, 78, 79, 83, 84, 89, 94, 99]	PE, PB, CE, MA, RN, BA

Alfazema	Lamiaceae	*Lavandula spica *Cav.	*Lavandula spica *Cav.	E	[1, 93]	PE
		*Hyptis suaveolens * (L.) Poit.	*Hyptis suaveolens * (L.) Poit.	N	[60]	CE
		*Lavandula officinalis *Chaix	*Lavandula officinalis *Chaix	E	[99]	RN
		*Hyptis pectinata * (L.) Poit.	*Hyptis pectinata * (L.) Poit.	N	[52]	PE
	Verbenaceae	*Aloysia lycioides *Cham.	*Aloysia lycioides *Cham.	N	[69]	BA

Alumã	Asteraceae	*Vernonia condensata* Baker *Vernonia bahiensis Toledo *	*Gymnanthemum amygdalinum * (Delile) Sch.Bip. ex Walp.	N	[67, 79, 83, 84, 101]	SE, BA

Babosa	Xanthorrhoeaceae	*Aloe vera * (L.) Burm. f. *Aloe barbadensis* Mill.	*Aloe vera* (L.) Burm. f.	E	[1, 3, 9, 11, 52, 53, 55–57, 66–69, 76, 78, 79, 81, 92–94, 99, 101]	PE, PB, SE, CE, PI, MA, RN, BA
		*Aloe socotrina * Schult. and Schult. f.	*Aloe socotrina * Schult. and Schult. f.	E	[83]	BA

Boldo	Monimiaceae	*Peumus boldus *Molina	*Peumus boldus *Molina	E	[1, 3, 52, 53, 63, 76, 81, 99, 100]	PE, PB, RN
	Lamiaceae	*Plectranthus barbatus *Andrews	*Plectranthus barbatus* Andrews	E	[57, 66, 69, 79]	PI, MA, BA
		*Coleus barbatus * (Andrews) Benth.			[79]	BA
		*Plectranthus neochilus *Schltr.	*Plectranthus neochilus *Schltr.	E	[67, 83]	BA

Cabacinha	Cucurbitaceae	*Luffa operculata * (L.) Cogn.	*Luffa operculata * (L.) Cogn.	N	[1, 3, 11, 53, 59, 62, 66, 68, 76, 77, 84, 88, 93]	PE, PB, MA, RN, BA

Camomila	Asteraceae	*Matricaria chamomila * L.	*Matricaria chamomila* L.	E	[3, 9, 11, 53, 67–69, 81, 84, 93]	PE, PB, CE, MA, RN, BA
		*Coreopsis grandiflora* Hogg ex Sweet	*Coreopsis grandiflora* Hogg ex Sweet	E	[94]	PE

Cana de macaco	Costaceae	*Costus spicatus * (Jacq.) Sw.	*Costus spicatus * (Jacq.) Sw.	N	[1, 78]	PE, CE
		*Costus spiralis * (Jacq.) Roscoe	*Costus spiralis * (Jacq.) Roscoe	N	[67, 94]	PE, BA
		*Costus arabicus* L.	*Costus arabicus* L.	N	[93]	PE

Canapum	Solanaceae	*Physalis angulata* L.	*Physalis angulata* L.	E	[57, 66, 68, 74]	PI, MA
	Passifloraceae	*Passiflora foetida* L.	*Passiflora foetida* L.	N	[70, 77]	PB

Caninana	Rubiaceae	*Chiococca alba* (L.) Hitchc.	*Chiococca alba* (L.) Hitchc.	N	[9, 82, 89]	PB, CE, BA
	Polygalaceae	*Polygala paniculata* L.	*Polygala paniculata* L.	N	[78]	CE

Capim santo	Poaceae	*Cymbopogon citratus * (DC.) Stapf	*Cymbopogon citratus * (DC.) Stapf	E	[1, 3, 9, 11, 52, 53, 55, 56, 63, 67, 69, 72, 75, 76, 78, 79, 83, 93, 94, 99–101]	PE, PB, SE, CE, RN, BA

Carambola	Oxalidaceae	*Averrhoa carambola* L.	*Averrhoa carambola* L.	E	[1, 11, 56, 57, 66, 79, 93, 94, 99]	PE, PI, MA, RN, BA

Carrapateira	Euphorbiaceae	*Ricinus communis* L.	*Ricinus communis* L.	E	[11, 56, 63, 71, 89, 93]	PE, PB

Cebola branca	Liliaceae	*Allium cepa* L.	*Allium cepa* L.	E	[3, 9, 92]	PB, CE
		Allium ascalonicum L.	Allium ascalonicum L.	E	[53, 55, 69, 76]	PE, CE, BA

Chambá	Acanthaceae	*Justicia pectoralis *Jacq.	*Justicia pectoralis *Jacq.	N	[53, 56, 65, 94]	PE

Colônia	Zingiberaceae	*Alpinia speciosa * (Blume) D. Dietr. *Alpinia zerumbet * (Pers.) B. L. Burtt and R. M. Sm.	*Alpinia speciosa * (Blume) D. Dietr.	E	[9, 53, 65, 76, 78, 84, 93, 94, 100]	PE, PB, CE, BA

Confrei	Boraginaceae	*Symphytum officinale* L.	*Symphytum officinale* L.	E	[53, 67, 69, 83, 84, 93, 94]	PE, BA

Cravo branco	Caryophyllaceae	*Dianthus caryophyllus* L.	*Dianthus caryophyllus* L.	E	[1, 11, 52, 53, 63, 65]	PE
	Asteraceae	*Tagetes erecta* L.	*Tagetes erecta* L.	E	[67, 72, 76, 93]	PE, PB

Erva moura	Solanaceae	*Solanum americanum *Mill.	*Solanum americanum *Mill.	E	[1, 11, 52, 53, 56, 57, 60, 76, 88]	PE, PB, CE, PI

Espinho de cigano	Asteraceae	*Acanthospermum hispidum *DC.	*Acanthospermum hispidum *DC.	E	[1, 3, 11, 52, 56, 63, 72, 75, 76, 88, 94, 100]	PE, PB

Eucalipto	Myrtaceae	*Eucalyptus globulus *Labill.	*Eucalyptus globulus *Labill.	E	[9, 57, 63, 67, 69, 71, 72, 79, 81, 84, 92]	PE, PB, CE, PI, RN, BA
		*Eucalyptus citriodora *Hook.	*Eucalyptus citriodora *Hook.	E	[11, 55, 56, 94]	PE, CE

Pinha	Annonaceae	*Annona squamosa* L.	*Annona squamosa* L.	E	[11, 56, 63, 75, 76, 84, 93, 99]	PE, RN, BA
		*Annona coriacea *Mart.	*Annona coriacea *Mart.	N	[98]	CE
		* Annona tomentosa *R. E. Fr.	* Annona tomentosa *R. E. Fr.	N	[98]	CE

Mamoeiro	Caricaceae	*Carica papaya* L.	*Carica papaya* L.	E	[3, 55, 68, 83, 92, 94]	PE, PB, CE, MA, BA

Gergelim	Pedaliaceae	*Sesamum orientale* L.	*Sesamum orientale* L.	E	[3, 9, 11, 53, 67, 81, 92]	PE, PB, CE, RN
		*Sesamum indicum* L.				

Girassol	Asteraceae	*Helianthus annuus* L.	*Helianthus annuus* L.	E	[9, 11, 53, 56, 63, 68, 69, 72, 92, 93]	PE, PB, CE, MA, BA
		*Tithonia diversifolia * (*Hemsl.*) A. Gray	*Tithonia diversifolia * (*Hemsl*.) A. Gray	E	[76]	PE

Imbira	Annonaceae	*Xylopia frutescens *Aubl.	*Xylopia frutescens *Aubl.	N	[53, 86]	PE, PB
		*Xylopia laevigata * (Mart.) R. E. Fr.	*Xylopia laevigata * (Mart.) R. E. Fr.	N	[89]	PB

Ipe	Bignoniaceae	*Tabebuia aurea* (Silva Manso) Benth. and Hook. f. ex S. Moore	*Tabebuia aurea* (Silva Manso) Benth. and Hook. f. ex S. Moore	N	[82]	BA
		*Tabebuia avellanedae * Lorentz ex Griseb.	*Handroanthus impetiginosus* (Mart. ex DC.) Mattos	N	[82]	BA
		*Tabebuia chrysotricha* (Mart. ex *A. *DC.) Standl.	*Handroanthus chrysotrichus* (Mart. ex DC.) Mattos	N	[82]	BA
		*Tabebuia roseo-alba * (Ridl.) Sandwith	*Tabebuia roseoalba* (Ridl.) Sandwith	N	[82]	BA

Pau d'arco roxo	Bignoniaceae	*Tabebuia avellanedae * Lorentz ex Griseb. *Tabebuia impetiginosa * (Mart. ex DC.) *Standl*.	*Handroanthus impetiginosus* (Mart. ex DC.) Mattos	N	[3, 9, 11, 54, 56, 58, 60, 65, 69, 70, 74, 85, 89, 100]	PE, PB, SE, CE, PI, BA

Pau d'arco	Bignoniaceae	*Tabebuia avellanedae *Lorentz ex Griseb.	*Handroanthus impetiginosus* (Mart. ex DC.) Mattos	N	[53, 62, 80]	PE, RN, BA
		*Tabebuia impetiginosa * (Mart. ex DC.) Standl.				
		*Tabebuia ochracea * (Cham.) Standl.	*Handroanthus ochraceus* (Cham.) Mattos	N	[93]	PE
		*Tabebuia serratifolia * (Vahl) G. Nicholson	*Handroanthus serratifolius* (A.H.Gentry) S. Grose	N	[74, 86]	PB, PI
		*Tabebuia spongiosa *Rizzini	*Handroanthus spongiosus* (Rizzini) S.Grose	N	[74]	PI
		*Tabebuia aurea* (Silva Manso) Benth. and Hook. f. ex S. Moore	*Tabebuia aurea* (Silva Manso) Benth. and Hook. f. ex S. Moore	N	[63, 92]	PE, CE
		*Tabebuia caraiba * (Mart.) Bureau				

Pepaconha	Violaceae	*Hybanthus ipecacuanha * (L.) Baill. *Hybanthus calceolaria * (L.) Oken	*Hybanthus calceolaria * (L.) Oken	N	[9, 53, 55, 56, 59, 71, 75, 77, 88, 90, 93, 94]	PE, PB, CE, RN
	Rubiaceae	*Psychotria ipecacuanha * (Brot.) Stokes *Cephaelis ipecacuanha * (Brot.) A. Rich.	*Carapichea ipecacuanha* (Brot.) L. Andersson	N	[1, 11, 81]	PE, RN

Losna	Asteraceae	*Artemisia absinthium* L.	*Artemisia absinthium* L.	E	[9, 54, 93]	PE, PB, CE
		*Artemisia vulgaris* L.	*Artemisia vulgaris* L.	E	[1, 65]	PE

Macassa	Lamiaceae	*Aeollanthus suaveolens *Mart. ex Spreng.	*Aeollanthus suaveolens *Mart. ex Spreng.	E	[53, 56, 63, 65, 76, 84]	PE, PB, BA

Jatobá	Fabaceae	*Hymenaea courbaril* L.	*Hymenaea courbaril* L.	N	[9, 53, 55, 61, 63, 66, 68, 71, 75, 76, 80, 85–87, 89, 92, 93, 100]	PE, PB, CE, PI, MA, BA
		*Hymenaea martiana *Hayne	*Hymenaea martiana *Hayne	N	[56]	PE
		*Hymenaea stigonocarpa *Mart. ex Hayne	*Hymenaea stigonocarpa *Mart. ex Hayne	N	[69, 98]	CE, BA
		*Hymenaea aurea *Y. T. Lee and Langenh.	*Hymenaea aurea *Y. T. Lee and Langenh.	N	[74]	PI

Jerimum	Cucurbitaceae	*Cucurbita pepo* L.	*Cucurbita pepo* L.	E	[11, 56, 68, 76, 93, 100]	PE, PB, MA
		*Cucurbita argyrosperma* Hort. ex L. H. Bailey	*Cucurbita argyrosperma* Hort. ex L. H. Bailey	E	[78]	CE

Hortelã miuda	Lamiaceae	*Coleus forskohlii* (Willd.) Briq.	*Coleus forskohlii* (Willd.) Briq.	E	[67]	PE
		*Mentha piperita* L.	*Mentha piperita* L.	E	[3]	PB
		*Mentha viridis * (L.) L.	*Mentha spicata* L.	E	[69]	BA

Hortelã grauda	Lamiaceae	*Plectranthus amboinicus * (Lour.) Spreng.	*Plectranthus unguentarius * Codd	E	[3, 53, 56, 69]	PE, PB, BA

Limão	Rutaceae	*Citrus aurantiifolia * (Christm.) Swingle	*Citrus aurantium* L.	E	[9]	CE
		*Citrus limonia * (L.) Osbeck	*Citrus medica* L.	E	[56, 66, 69, 78, 79, 81, 84, 92, 99]	PE, CE, MA, RN, BA
		*Citrus limon * (L.) Osbeck				
		*Citrus limonum *Risso				

Macela	Arecaceae	*Egletes viscosa* (L.) Less.	*Egletes viscosa* (L.) Less.	E	[1, 3, 9, 11, 53, 59, 60, 67, 72, 75, 78, 84, 88, 101]	PE, PB, SE, CE, RN, BA
	Asteraceae	*Achyrocline satureioides * (Lam.) DC.	*Achyrocline satureioides * (Lam.) DC.	N	[81, 99]	RN
	Lamiaceae	*Hyptis martiusii *Benth.	*Hyptis martiusii *Benth.	N	[80]	BA

Malicia	Fabaceae	*Mimosa invisa *Mart. ex Colla	*Mimosa invisa *Mart. ex Colla	N	[56]	PE
		Schrank*ia leptocarpa *DC.	*Mimosa candollei* R. Grether	N	[56]	PE
		*Mimosa misera *Benth.	*Mimosa misera *Benth.	N	[90]	RN
		*Mimosa somnians *Humb. and Bonpl. ex Willd.	*Mimosa somnians *Humb. and Bonpl. ex Willd.	N	[85]	PB
		*Mimosa pudica* L.	*Mimosa pudica* L.	N	[78]	CE

Malva	Sterculiaceae	*Piriqueta racemosa * (Jacq.) Sweet	*Piriqueta racemosa * (Jacq.) Sweet	N	[95]	SE
		*Melochia tomentosa* L.	*Melochia tomentosa* L.	N	[75]	PE
		*Waltheria indica* L.	*Waltheria americana* L.	N	[78]	CE
		*Piriqueta guianensis *N. E. Br.	*Piriqueta guianensis *N. E. Br.	N	[95]	SE
	Lamiaceae	*Plectranthus barbatus *Andrews	*Plectranthus barbatus *Andrews	E	[55]	CE
	Malvaceae	*Malva sylvestris* L.	*Malva erecta* J. Presl and C. Presl	E	[81, 99]	RN
		*Sida linifolia *Cav.	*Sida linifolia *Cav.	N	[89]	PB

Manga espada	Anacardiaceae	*Mangifera indica* L.	*Mangifera indica* L.	E	[52, 69, 76]	PE

Capitãozinho	Gomphrenaceae	*Gomphrena demissa *Mart.	*Gomphrena demissa *Mart.	N	[54, 59, 62, 71, 77]	PB, RN

Malva rosa	Sterculiaceae	*Melochia tomentosa* L.	*Melochia tomentosa* L.	N	[3]	PB
	Geraniaceae	*Geranium erodifolium* L.	*Geranium erodifolium* L.	E	[53]	PE
	Malvaceae	*Alcea rosea* L.	*Althaea rosea* (L.) Cav.	E	[100]	PB
		*Urena lobata* L.	*Urena lobata* L.	N	[1, 11, 93]	PE

Malva branca	Sterculiaceae	*Waltheria rotundifolia *Schrank	*Waltheria rotundifolia *Schrank	N	[3]	PB
	Malvaceae	*Sida cordifolia* L.	*Sida cordifolia* L.	N	[1, 57, 60, 67, 69, 88]	PE, PB, CE, PI, BA
		*Sida galheirensis *Ulbr.	* Sida galheirensis *Ulbr.	N	[77]	PB

Manjericão	Lamiaceae	*Ocimum basilicum* L.	*Ocimum basilicum* L.	E	[3, 53, 55, 65, 67, 69, 72, 76, 78, 79, 81, 94, 101]	PE, PB, SE, CE, RN, BA
		*Ociumum americanum* L.	*Ocumum americanum* L.	E	[57, 78, 83, 93]	PE, CE, PI, BA
		*Ocimum minimum* L.	*Ocimum minimum* L.	E	[68]	MA
		*Ocimum sanctum* L.	*Ocimum tenuiflorum* L.	E	[84]	BA

Mastruz	Chenopodiaceae	*Chenopodium ambrosioides* L.	*Chenopodium ambrosioides* L.	E	[1, 3, 9, 11, 52, 53, 55–57, 59, 61–63, 66–69, 72, 75, 76, 79–81, 83, 92–94, 100]	PE, PB, CE, PI, MA, RN, BA

Melão de São Caetano	Cucurbitaceae	*Momordica charantia* L.	*Momordica charantia* L.	E	[53, 55–57, 60, 66, 72, 75, 76, 79, 83–85, 90, 92, 94, 99]	PE, PB, CE, PI, MA, RN, BA

Mufumbo	Combretaceae	*Combretum fruticosum * (Loefl.) Stuntz	*Combretum fruticosum * (Loefl.) Stuntz	N	[57, 70]	PB, PI
		*Combretum leprosum *Mart.	*Combretum leprosum *Mart.	N	[62, 71, 90]	PB, RN
		*Combretum mellifluum *Eichler	*Combretum mellifluum *Eichler	N	[61]	MA

Mutamba	Sterculiaceae	*Guazuma ulmifolia *Lam.	*Guazuma ulmifolia *Lam.	N	[1, 11, 54, 56, 66, 69, 82, 85, 86, 94]	PE, PB, MA, BA
	Ulmaceae	*Trema micrantha * (L.) Blume	*Trema micrantha * (L.) Blume	N	[74]	PI

Pereiro	Apocynaccae	*Aspidosperma parvifolium *A. DC.	*Aspidosperma parvifolium *A. DC.	N	[94]	PE
		*Aspidosperma pyrifolium *Mart.	*Aspidosperma pyrifolium *Mart.	N	[3, 52, 53, 58, 59, 62, 63, 70, 73–77, 80, 88, 95]	PE, PB, SE, PI, RN, BA
	Tiliaceae	*Luehea ochrophylla *Mart.	*Luehea ochrophylla *Mart.	N	[56]	PE

Pega pinto	Nyctaginaceae	*Boerhavia diffusa* L.	*Boerhavia diffusa* L.	E	[1, 3, 9, 11, 52, 53, 57, 59, 61, 63, 75, 76, 78, 90, 93–95]	PE, PB, SE, CE, PI, MA, RN
		*Boerhavia coccinea* Mill.	*Boerhavia coccinea* Mill.	E	[55, 60, 69]	CE, BA
		*Boerhavia hirsuta *Jacq.				

Pitanga	Myrtaceae	*Eugenia uniflora* L.	*Eugenia uniflora* L.	N	[11, 52, 56, 63, 67, 69, 75, 76, 78, 79, 83, 93, 94]	PE, CE, BA
		*Eugenia pitanga * (O. Berg) Kiaersk.	*Eugenia pluriflora *DC.	N	[84]	BA

Pinhão roxo	Euphorbiaceae	*Jatropha gossypiifolia* L.	*Jatropha gossypiifolia* L.	N	[56, 57, 69, 71, 75, 76, 78, 99]	PE, PB, CE, PI, RN, BA
		*Jatropha ribifolia * (Pohl) Baill.	*Jatropha ribifolia * (Pohl) Baill.	N	[80]	BA

Poejo	Lamiaceae	*Mentha pulegium* L.	*Mentha pulegium* L.	E	[53, 67, 83, 93]	PE, BA

Quebra faca	Euphorbiaceae	*Croton conduplicatus *Kunth	*Croton conduplicatus *Kunth	N	[9]	CE
		*Croton rhamnifolius * Willd.	*Croton heliotropiifolius* Kunth	N	[53]	PE
		*Croton cordiifolius* Baill.	*Croton cordiifolius* Baill.	N	[60]	CE

Quiabo	Malvaceae	*Hibiscus esculentus* L.	*Abelmoschus esculentus* (L.) Moench	E	[53, 55, 56, 66, 67, 76, 78, 84]	PE, CE, MA, BA
		*Abelmoschus esculentus* (L.) Moench				

Quina	Rubiaceae	*Coutarea hexandra * (Jacq.) K. Schum.	*Coutarea hexandra * (Jacq.) K. Schum.	N	[9, 53, 58, 69, 71, 76, 86, 94]	PE, PB, SE, CE, BA
		*Cinchona calisaya* Wedd.	*Cinchona officinalis* L.	N	[66]	MA
	Simaroubaceae	*Quassia amara* L.	*Quassia amara* L.	N	[68]	MA
	Rubiaceae	*Chiococca brachiata *Ruiz and Pav.	*Chiococca alba* (L.) Hitchc.	N	[80]	BA

Romã	Punicaceae	*Punica granatum* L.	*Punica granatum* L.	E	[3, 9, 53–55, 57, 61, 63, 65, 68, 69, 72, 76, 78, 79, 81, 83, 93, 99, 100]	PE, PB, CE, PI, MA, RN, BA
	Brassicaceae	*Armoracia rusticana * G. Gaertn., B. Mey., and Scherb.	*Armoracia rusticana * G. Gaertn., B. Mey., and Scherb.	E	[84]	BA

Saião	Crassulaceae	*Kalanchoe brasiliensis *Cambess.	*Kalanchoe brasiliensis *Cambess.	E	[3, 53, 72]	PE, PB

Salsa	Convolvulaceae	*Ipomoea asarifolia * (*Desr.*) Roem. and Schult.	*Ipomoea asarifolia * (*Desr.*) Roem. and Schult.	N	[11, 56, 57, 59, 60, 65, 78, 85, 89]	PE, PB, CE, PI, RN
		*Ipomoea pes-caprae * (L.) R. Br.	*Ipomoea pes-caprae * (L.) R. Br.	N	[95]	SE
	Apiaceae	*Petroselinum crispum* (Mill.) Fuss	*Petroselinum crispum* (Mill.) Fuss	E	[67]	BA
		*Petroselinum sativum* Hoffm.	*Petroselinum sativum* Hoffm.	E	[84]	BA

Sambacaitá	Lamiaceae	*Hyptis pectinata * (L.) Poit.	*Hyptis pectinata * (L.) Poit.	N	[58, 80, 93]	PE, SE, BA
		*Hyptis suaveolens * (L.) Poit.	*Hyptis suaveolens * (L.) Poit.	N	[94]	PE
		*Hyptis mutabilis * (Rich.) Briq.	*Hyptis mutabilis * (Rich.) Briq.	N	[76]	PE

Sena	Fabaceae	*Senna acutifolia *Link	*Senna alexandrina* Mill.	N	[53]	PE
		*Senna corymbosa* (Lam.) H. S. Irwin and Barneby	*Senna corymbosa* (Lam.) H. S. Irwin and Barneby	N	[94]	PE
		*Senna martiana* (Benth.) H. S. Irwin and Barneby	*Senna martiana* (Benth.) H. S. Irwin and Barneby	N	[77]	PB
		*Tephrosia purpurea* (L.) Pers.	*Tephrosia purpurea* (L.) Pers.	N	[69]	BA

Sucupira	Fabaceae	*Bowdichia virgilioides *Kunth	*Bowdichia virgilioides *Kunth	N	[1, 11, 56, 76, 86, 89, 98]	PE, PB, CE
		*Bowdichia nitida *Spruce ex Benth.	*Bowdichia nitida *Spruce ex Benth.	N	[68]	MA

Tamarino	Fabaceae	*Tamarindus indica* L.	*Tamarindus indica* L.	E	[9, 11, 56, 57, 61, 63, 72, 83, 84, 92, 93]	PE, PB, CE, PI, MA, BA

Guiné	Phytolacacceae	*Petiveria alliacea* L.	*Petiveria alliacea* L.	N	[53, 72, 84]	PE, PB, BA
		*Petiveria tetrandra* B. A. Gomes	*Petiveria tetrandra* B. A. Gomes	N	[79]	BA

Urucum	Bixaceae	*Bixa orellana* L.	*Bixa orellana* L.	N	[9, 55, 56, 61, 66, 69, 78, 81, 83, 84, 92]	PE, CE, MA, RN, BA

Tiririca	Cyperaceae	*Cyperus ligularis* L.	*Cyperus ligularis* L.	N	[95]	SE
		*Cyperus surinamensis *Rottb.	*Cyperus surinamensis *Rottb.	N	[95]	SE
		*Fimbristylis dichotoma * (L.) Vahl	*Fimbristylis dichotoma * (L.) Vahl	N	[95]	SE
		*Fimbristylis littoralis *Gaudich.	*Fimbristylis miliacea* (L.) Vahl	N	[95]	SE

Junco	Cyperaceae	*Eleocharis interstincta * (Vahl) Roem. and Schult.	*Eleocharis interstincta * (Vahl) Roem. and Schult.	N	[56]	PE
		*Eleocharis elegans * (Kunth) Roem. and Schult.	*Eleocharis elegans * (Kunth) Roem. and Schult.	N	[60]	CE
		*Cyperus articulatus* L.	*Cyperus articulatus* L.	N	[59]	RN
		*Cyperus esculentus* L.	*Cyperus esculentus* L.	N	[54]	PB

Tomate	Solanaceae	*Lycopersicon esculentum * MilL.	*Solanum lycopersicum* L.	E	[56, 57, 67, 93, 99]	PE, PI, RN
		*Physalis ixocarpa *Brot. ex Hornem.	*Physalis philadelphica* Lam.	E	[68]	MA

Trapiá	Capparidaceae	*Crataeva tapia* L.	*Crataeva tapia* L.	E	[53, 57, 63, 66, 70, 73, 75]	PE, PB, PI, MA

Urtiga branca	Euphorbiaceae	*Cnidoscolus urens * (L.) Arthur	*Cnidoscolus urens * (L.) Arthur	N	[1, 11, 53, 56, 59, 71]	PE, PB, RN
		*Cnidoscolus phyllacanthus * (Mull. Arg.) Pax and L. Hoffm.	*Cnidoscolus phyllacanthus * (Mull. Arg.) Pax and L. Hoffm.	N	[76]	PE
		*Cnidoscolus infestus *Pax and K. Hoffm.	*Cnidoscolus infestus *Pax and K. Hoffm.	N	[77]	PB
	Lamiaceae	*Lamium album* L.	*Lamium album* L.	E	[100]	PB
	Loasaceae	*Aosa rupestris * (Gardner) Weigend	*Aosa rupestris * (Gardner) Weigend	N	[77]	PB
	Urticaceae	*Urtica urens* L.	*Urtica urens* L.	E	[54]	PB

Jurema branca	Fabaceae	*Piptadenia stipulacea * (Benth.) Ducke	*Piptadenia stipulacea * (Benth.) Ducke	N	[3, 11, 60, 90]	PE, PB, CE, RN
		*Senegalia piauhiensis * (Benth.) Seigler and Ebinger	*Senegalia piauhiensis * (Benth.) Seigler and Ebinger	N	[58]	SE
		*Calliandra spinosa *Ducke	*Calliandra spinosa *Ducke	N	[90]	RN
		*Mimosa ophthalmocentra *Mart. ex Benth.	*Mimosa ophthalmocentra * Mart. ex Benth.	N	[85]	PB
		*Mimosa tenuiflora * (Willd.) Poir.	*Mimosa tenuiflora * (Willd.) Poir.	N	[95]	SE
		*Acacia farnesiana * (L.) Willd.	*Vachellia farnesiana * (L.) Wight and Arn.	N	[63, 73]	PE

Jurema preta	Fabaceae	*Mimosa tenuiflora * (Willd.) Poir.	*Mimosa tenuiflora * (Willd.) Poir.	N	[1, 3, 9, 11, 52, 53, 58–60, 63, 70–73, 75, 79–81, 85, 93, 95]	PE, PB, SE, CE, RN, BA
		*Mimosa acutistipula * (Mart.) Benth.	*Mimosa acutistipula * (Mart.) Benth.	N	[54]	PB

Jurubeba branca	Solanaceae	*Solanum rhytidoandrum Sendtn. *	*Solanum rhytidoandrum Sendtn. *	N	[58, 77, 88]	PB, SE
		*Solanum polytr*ich*um *Moric.	*Solanum polyt*rich*um *Moric.	N	[82]	BA
		*Solanum stipulaceum *Roem. and Schult.	*Solanum stipulaceum *Roem. and Schult.	N	[60]	CE
		*Solanum albidum* Dunal	*Solanum albidum* Dunal	E	[55]	CE

Imburana de cheiro	Fabaceae	*Amburana cearensis * (Allemão) A. C. Sm.	*Amburana cearensis * (Allemão) A. C. Sm.	N	[1, 52, 57, 60, 63, 67, 72, 76, 77, 92]	PE, PB, CE, PI
	Anacardiaceae	*Myracrodruon urundeuva* Allemão	*Myracrodruon urundeuva* Allemão	N	[53]	PE

PE: Pernambuco, PB: Paraiba. SE: Sergipe, CE: Ceará. RN: Rio Grande do Norte, BA: Bahia, MA: Maranhão, PI: Piauí.

## References

[1] Albuquerque UP, Monteiro JM, Ramos MA, de Amorim ELC (2007). Medicinal and magic plants from a public market in northeastern Brazil. *Journal of Ethnopharmacology*.

[2] Pinto AC, Silva DH, Bolzani VS, Lopes NP, Epifanio RA (2002). Produtos naturais: atualidade, desafios e perspectivas. *Química Nova*.

[3] Veeman M, Campbell BM, Luckert MK (2002). Conociendo los mercados locales y regionales para produtos forestales. *Evaluando la Cosecha Oculta de los Bosques*.

[4] Cunningham AB (2001). *Applied Ethnobotany—People, Wild Plant Use & Conservation*.

[5] Hanlidou E, Karousou R, Kleftoyanni V, Kokkini S (2004). The herbal market of Thessaloniki (N Greece) and its relation to the ethnobotanical tradition. *Journal of Ethnopharmacology*.

[6] Krog M, Falcão M, Olsen CS (2006). Medicinal plants markets and trade in Maputo, Mozambique. *Forest e Landscape Working Papers*.

[7] Monteiro JM, Ramos MA, Araújo EL, Amorim ELC, Albuquerque UP (2011). Collection and commerce of the Myracrodruon urundeuva Allemão bark in the semi-arid region of Northeastern Brazil. *Bioremediation, Biodiversity & Bioavailability*.

[8] Almeida CDFCBR, de Amorim ELC, de Albuquerque UP, Maia MBS (2006). Medicinal plants popularly used in the Xingó region—a semi-arid location in Northeastern Brazil. *Journal of Ethnobiology and Ethnomedicine*.

[9] Cartaxo SL, Souza MMM, de Albuquerque UP (2010). Medicinal plants with bioprospecting potential used in semi-arid northeastern Brazil. *Journal of Ethnopharmacology*.

[10] Bieski IGC, Santos FR, de Oliveira RM (2012). Ethnopharmacology of medicinal plants of the pantanal region (Mato Grosso, Brazil). *Evidence-Based Complementary and Alternative Medicine*.

[11] de Almeida CDFCBR, Ramos MA, Silva RRV (2012). Intracultural variation in the knowledge of medicinal plants in an urban-rural community in the Atlantic Forest from Northeastern Brazil. *Evidence-Based Complementary and Alternative Medicine*.

[12] Lev E, Amar Z (2002). Ethnopharmacological survey of traditional drugs sold in the Kingdom of Jordan. *Journal of Ethnopharmacology*.

[13] Ramos MA, Albuquerque UP, Amorim ELC, Albuquerque UP, Almeida CFCBR, Marins JFA (2005). O comércio de plantas medicinais em mercados públicos e feiras livres: um estudo de caso. *Tópicos em conservação, etnobotânica e etnofarmacologia de plantas medicinais e mágicas*.

[14] Molares S, Ladio A, Albuquerque UP, Lucena RFP, Cunha LVFC (2010). Métodos micrográficos aplicados à pesquisa etnobotânica. *Métodos e Técnicas na Pesquisa Etnobiológica e Etnoecológica*.

[15] Li M, Cao H, But PP-H, Shaw P-C (2011). Identification of herbal medicinal materials using DNA barcodes. *Journal of Systematics and Evolution*.

[16] Nguyen MLT (2005). Cultivated plant collections from markets places. *Ethnobotany Research e Applications*.

[17] Lee S, Xiao C, Pei S (2008). Ethnobotanical survey of medicinal plants at periodic markets of Honghe Prefecture in Yunnan Province, SW China. *Journal of Ethnopharmacology*.

[18] Conklin HC (1962). Lexicographical treatment of folk taxonomies. *Journal of American Linguistics*.

[19] Metzger DG, Williams GE (1966). Some procedurs and results in the study of native categories: tzeltal ‘firewood’. *American Anthropologist*.

[20] Berlin B, Breedlove DE, Raven PH (1968). Covert categories and folk taxonomies. *American Anthropologist*.

[21] Hartmann T (1967). *A Nomenclatura Botânica Borôro*.

[22] Berlin B (1973). Folk systematics in relationsystematics in relation to biological classification and nomenclature. *Annual Review of Ecology and Systematics*.

[23] Berlin B (1992). *Ethnobiological Classification: Principles of Categorization of Plants and Animals in Traditional Societies*.

[24] Vendruscolo GS, Eisinger SM, Soares EC, Zachia RA (2005). Estudo etnobotânico do uso dos recursos vegetais em São João do Polêsine, RS, Brasil, no período de outubro de 1999 a junho de 2073. II—Etnotaxonomia: critérios taxonômicos e sistema de classificação folk. *Revista Brasileira de Plantas Medicinais, Botucatu*.

[25] Hiepko P (2006). Eipo plant nomenclature and classification compared with other folk taxonomic systems. *Willdenowia*.

[26] Haverroth M (2007). *Etnobotânica, uso e classificação dos vegetais pelos Kaingang*.

[27] Khasbagan K, Soyolt S (2008). Indigenous knowledge for plant species diversity: a case study of wild plants’ folk names used by the Mongolians in Ejina desert area, Inner Mongolia, P. R. China. *Journal of Ethnobiology and Ethnomedicine*.

[28] Signorini MA, Piredda M, Bruschi P (2009). Plants and traditional knowledge: an ethnobotanical investigation on Monte Ortobene (Nuoro, Sardinia). *Journal of Ethnobiology and Ethnomedicine*.

[29] Burke P (2003). *Hibridismo Cultural*.

[30] Nesheim I, Dhillion SS, Stølen KA (2006). What happens to traditional knowledge and use of natural resources when people migrate?. *Human Ecology*.

[31] International Institute for Enviromental and Development (IIED) (1995). *The Hidden Haverst: The Value of Wild Resources in Agricultural Systems*.

[32] International Institute for Enviromental and Development (IIED) Valuing the hidden haverst: methodological approaches for local level economic analysis of wild resources.

[33] Elisabetsky E, Wannmacher L (1993). The status of ethnopharmacology in Brazil. *Journal of Ethnopharmacology*.

[34] IBGE Instituto Brasileiro de Geografia e Estatística. http://www.ibge.gov.br/.

[35] Leal IR, Tabarelli M, Silva JMC (2003). *Ecologia e conservação da Caatinga*.

[36] Prado D, Leal IR, Tabarelli M, Silva JMC (2003). As caatingas da América do Sul. *Ecologia e conservação da Caatinga*.

[37] Giulietti AM, Bocage Neta AL, Castro AAJF, Silva JMC, Tabarelli M, Fonseca MT, Lins LV (2004). Diagnóstico da vegetação nativa do bioma Caatinga. *Biodiversidade da Caatinga: áreas e ações prioritárias para a conservação*.

[38] Myers N, Mittermeler RA, Mittermeler CG, da Fonseca GAB, Kent J (2000). Biodiversity hotspots for conservation priorities. *Nature*.

[39] Galindo-Leal C, Câmara IG, Galindo-Leal C, Câmara IG (2003). Atlantic forest hotspots status: an overview. *The Atlantic Forest of South America: Biodiversity Status, Threats, and Outlook*.

[40] Rizzini CT (1997). *Tratado de Fitogeografia de Brasil: Aspectos ecológicos, sociológicos e florísticos*.

[41] Castro AAJF, Martins FR (1999). Cerrados do Brasil e do Nordeste: caracterização, área de ocupação e considerações sobre a sua fitodiversidade. *Pesquisa em Foco*.

[42] Pennington RT, Lavin M, Prado DE, Pendry CA, Pell SK, Butterworth CA (2004). Historical climate change and speciation: Neotropical seasonally dry forest plants show patterns of both Tertiary and Quaternary diversification. *Philosophical Transactions of the Royal Society B*.

[43] Tabarelli M, Santos AMM, Porto KC, Cabral JJP, Tabarelli M (2004). Uma breve descrição sobre a história natural dos Brejos Nordestinos. *Brejos de Altitude em Pernambuco e Paraíba, História Natural, Ecologia e Conservação*.

[44] Tabarelli M, Pinto SRR, Leal IR (2009). Floresta Atlântica nordestina: fragmentação, degeneração e conservação. *Ciência Hoje*.

[45] Leal IR, Silva JM, Tabarelli M, Lacher TE (2005). Mudando o curso da conservação da biodiversidade na Caatinga do Nordeste do Brasil. *Megadiversidade*.

[46] Freyre G (1989). *Nordeste*.

[47] Ribeiro D (1995). *O Povo Brasileiro: A Formação e o Sentido de Brasil*.

[48] Martin GJ (1995). *Ethnobotany, A Methods Manual*.

[49] Jardim Botânico do Rio de Janeiro Lista de Espécies da Flora do Brasil 2012. http://floradobrasil.jbrj.gov.br/.

[50] Missouri Botanical Garden (MOBOT)—W3 TROPICOS. http://www.mobot.org/.

[51] Sokal RR, Rholf FG (1995). *Biometry*.

[52] Albuquerque UPD, Oliveira RFD (2007). Is the use-impact on native caatinga species in Brazil reduced by the high species richness of medicinal plants?. *Journal of Ethnopharmacology*.

[53] Monteiro JM, Ramos MA, Araújo EDL, Amorim ELC, Albuquerque UP (2011). Dynamics of medicinal plants knowledge and commerce in an urban ecosystem (Pernambuco, Northeast Brazil). *Environmental Monitoring and Assessment*.

[54] Agra CA (2007). Identificação das plantas medicinais indicadas pelos raizeiros e utilizados pelas mulheres no combate a enfermidades do aparelho geniturinário da cidade de campina grande-PB. *BIOFAR*.

[55] Morais SM, Dantas JDP, Silva ARA, Mangalhães EF (2005). Plantas medicinais usadas pelos índios Tapebas do Ceará. *Revista Brasileira de Farmacognosia*.

[56] Silva AJR, Andrade LHC (2005). Etnobotânica Nordestina: estudo comparativo da relação entre comunidades e vegetação na Zona do Litoral- Mata do Estado de Pernambuco, Brasil. *Acta Botanica Brasílica*.

[57] Oliveira FCS, Barros RFM, Moita Neto JM (2010). Plantas medicinais utilizadas em comunidades rurais de Oeiras, semiárido piauiense. *Revista Brasileira de Plantas Medicinais*.

[58] Machado WJ, Prata APN, Mello AA (2012). Floristic composition in areas of caatinga and brejo de altitude in sergipe state, Brazil. *Check List*.

[59] Roque AA, Rocha RM, Loiola MIB (2010). Uso e diversidade de plantas medicinais da Caatinga na comunidade rural de Laginhas, município de Caicó, Rio Grande do Norte (Nordeste do Brasil). *Revista Brasileira de Plantas Medicinais*.

[60] Medeiros JBLP (2004). *Zoneamento Fito-Ecológico da Estação Ecológica de Aiuaba: Uma Contribuição à Educação Ambiental e à Pesquisa Científica, [M.S. thesis]*.

[61] Nascimento JM, Conceiçao GM (2011). Plantas Medicinais e indicações Terapêuticas da Comunidade Quilombola Olho D’água do Raposo, Caxias, Maranhão, Brasil. *Revista de Biologia e Farmácia*.

[62] Silva MS, Antoniolli AR, Batista JS, Mota CN (2006). Plantas medicinais usadas nos distúrbios do trato gastrintestinal no povoado Colônia Treze Lagarto, SE, Brasil. *Acta Botanica Brasílica*.

[63] de Albuquerque UP, de Sousa Araújo TA, Ramos MA (2009). How ethnobotany can aid biodiversity conservation: reflections on investigations in the semi-arid region of NE Brazil. *Biodiversity and Conservation*.

[64] de Oliveira RLC, Lins Neto EMF, Araújo EL, Albuquerque UP (2007). Conservation priorities and population structure of woody medicinal plants in an area of caatinga vegetation (Pernambuco State, NE Brazil). *Environmental Monitoring and Assessment*.

[65] Albuquerque UP (1997). Plantas Medicinais e Mágicas Comercializadas Nos Mercados Públicos do Recife-PE. *Ciência e Trópico*.

[66] Monteles R, Pinheiro CUB (2007). Plantas medicinais em um quilombo maranhense: uma perspectiva etnobotânica. *Revista de Biologia e Ciências da Terra*.

[67] Silva FDS, Ramos MA, Hanazaki N, de Albuquerque UP (2011). Dynamics of traditional knowledge of medicinal plants in a rural community in the Brazilian semi-arid region. *Brazilian Journal of Pharmacognosy*.

[68] Madaleno IM (2011). Plantas da medicina popular de São Luís, Brasil. *Boletim do Museu Paraense Emilio Goeldi. Ciencias Humanas*.

[69] Barboza da Silva NC, Delfino Regis AC, Esquibel MA, Espírito Santo Santos J, Almeida MZ (2012). Uso de plantas medicinais na comunidade quilombola da Barra II—Bahia, Brasil. *Boletín Latinoamericano y del Caribe de Plantas Medicinales y Aromáticas*.

[70] Leite AP, Pedrosa KM, Lucena CM, Carvalho TKN, Felix LP, Lucena RFP (2012). Uso e conhecimento de espécies vegetais úteis em uma comunidade rural no vale do Piancó (Paraíba, Nordeste, Brasil). *Biofar: Revista de Biologia e Farmácia*.

[71] Marinho MGV, Silva CC, Andrade LHC (2011). Levantamento etnobotânico de plantas medicinais em área de caatinga no município de São José de Espinharas, Paraíba, Brasil. *Revista Brasileira de Plantas Medicinais*.

[72] Araujo MM (2009). *Estudo etnobotânico das plantas utilizadas como medicinais no assentamento Santo Antônio, Cajazeiras, PB, Brasil [M.S. thesis]*.

[73] Lucena RFP, Albuquerque UP, Monteiro JM, Almeida CDFBR, Florentino ATN, Ferraz JSF (2007). Useful plants of the semi-arid northeastern region of Brazil—a look at their conservation and sustainable use. *Environmental Monitoring and Assessment*.

[74] Lemos JR (2004). Composição florística do Parque Nacional Serra da Capivara, Piauí, Brasil. *Rodriguesia*.

[75] Albuquerque UP, Andrade LHC (2002). Conhecimento botânico tradicional e conservação em uma área de caatinga no estado de Pernambuco. *Acta Botanica Brasilica*.

[76] de Albuquerque UP, da Silva VA, Cabral MDC, Leal Alencar N, Andrade LDHC (2008). Comparisons between the use of medicinal plants in indigenous and rural caatinga (dryland) communities in NE Brazil. *Boletin Latinoamericano y del Caribe de Plantas Medicinales y Aromáticas*.

[77] Agra MF, Baracho GS, Nurit K, Basílio IJLD, Coelho VPM, Barbosa DA (2007). Sinopse da flora medicinal do Cariri Paraibano. *Oecologia Brasiliensis*.

[78] Balcazar AL (2012). *Hipótese da aparência na dinâmica do uso de plantas medicinais na floresta nacional do Araripe (Ceará, Noredeste do Brasil) [M.S. thesis]*.

[79] Castro JA, Brasileiro BP, Lyra DH, de Almeida Pereira D, Chaves JL, Amaral CLF (2011). Ethnobotanical study of traditional uses of medicinal plants: the flora of caatinga in the community of Cravolândia-BA, Brazil. *Journal of Medicinal Plant Research*.

[80] Almeida VS, Bandeira FPSF (2010). O significado cultural do uso de plantas da caatinga pelos quilombolas do Raso da Catarina, município de Jeremoabo, Bahia, Brasil. *Rodriguesia*.

[81] Guerra AMNM, Pessoa MF, Souza CSM, Maracajá PB (2010). Utilização de plantas medicinais pela comunidade rural Moacir Lucena, Apodi-RN. *Bioscience Journal*.

[82] Brito RC (2008). *Estudo Preliminar de Avaliação Ambiental Estratégica do Plano Diretor—Campus Ondina*.

[83] Costa LCB, Moreira RCT, Costa RCS, Lucena EARM (2002). Abordagem etnobotânica acerca do uso de plantas medicinais na Vila Cachoeira, Ilhéus, Bahia, Brasil. *Acta Farmaceutica Bonaerense*.

[84] Almeida MZ (2011). *Plantas Medicinais*.

[85] Abreu DBO, Oliveira Filho RB, Vasconcelos Netos CFA, Lucena CM, Felix LP, Lucena RFP (2011). Classificação etnobotânica por uma comunidade rural em um brejo de altitude no Nordeste do Brasil. *Biofar: Revista de Biologia e Farmácia*.

[86] Oliveira FX, Andrade LA, Félix LP (2006). Comparações florísticas e estruturais entre comunidades de Floresta Ombrófila Aberta com diferentes idades, no Município de Areia, PB, Brasil. *Acta Botanica Brasílica*.

[87] Matos MQ, Felili JM (2010). Florística, fitossociologia e diversidade da vegetação arbórea nas matas de galeria do Parque Nacional de Sete Cidades (PNSC), Piauí, Brasil. *Acta Botancia Brasilica*.

[88] Agra MF, Baracho GS, Nurit K, Basílio IJLD, Coelho VPM (2007). Medicinal and poisonous diversity of the flora of “Cariri Paraibano”, Brazil. *Journal of Ethnopharmacology*.

[89] Pereira MS, Alves RRN (2006). Composição florística de um remanescente de Mata Atlântica na Área de Proteção Ambiental Barra do Rio Mamanguape, Paraíba, Brasil. *Revista de Biologia e Ciências da Terra*.

[90] Loiola MIB, Paterno GBDC, Diniz JA, Calado JF, de OLiveira ACP (2010). Leguminosae and its potencial of use in the rural communities of São miguel do gostoso—RN. *Revista Caatinga*.

[91] Silva PMS, Brandão DO, Chaves TP (2012). Study bioprospecting of medicinal plant extracts of the semi-arid northeast: contribution to the control of oral microorganisms. *Evidence-Based Complementary and Alternative Medicine*.

[92] Oliveira IG, Cartaxo LS, da Silva MAP (2007). Plantas medicinais utilizadas na farmacopéia popular em Crato, Juazeiro e Barbalha (Ceará, Brasil). *Revista Brasileira de Biociências*.

[93] Texeira SA, Melo JIM (2006). Plantas medicinais utilizadas no município de Jupi, Pernambuco, Brasil. *Iheringiano*.

[94] Oliveira GL (2007). *Etnobotânica nordestina: plantas medicinais utilizadas na comunidade Muribeca (Jaboatão dos Guararapes, PE) [M.S. thesis]*.

[95] Silva ACC (2010). *Monumento Natural Grota do Angico, Sergipe, Brasil: Florística, estrutura da vegetação e conservação [M.S. thesis]*.

[96] Santos CS (2009). *Diagnóstico da flora apícola para sustentabildade da apicultura no Estado de Sergipe [M.S. thesis]*.

[97] Omena MLRA (2003). *Estudo etnofarmacológico de plantas com ação no sistema nervoso central: perspectiva de sustentabilidade em Umbuzeiro do Matuto—Porto da Folha/SE [M.S. thesis]*.

[98] Costa IR, Araújo FS, Lima-Verde LW (2004). Flora e aspectos auto-ecológicos de um encrave de cerrado na chapada do Araripe, Nordeste do Brasil. *Acta Botanica Brasilica*.

[99] Morais VM *Etnobotânica nos quintais da comunidade de Abderramant em Caraúbas—RN [Ph.D. thesis]*.

[100] Sales GPS, Albuquerque HN, Cavalcanti MLF (2004). Estudo do uso de plantas medicinais pela comunidade quilombola Senhor do Bonfim, Areia (PB). *Revista de Biologia e Ciências da Terra*.

[101] Silva TS, Freire EMX (2010). Abordagem etnobotânica sobre plantas medicinais citadas por populações do entorno de uma unidade de conservação da caatinga do Rio Grande do Norte, Brasil. *Revista Brasileira de Plantas Medicinais*.

[102] Judd WS, Campbell CS, Kellog EA, Stevens PF, Donoghue MJ (2009). *Sistemática Vegetal: Um Enfoque Filogenético*.

[103] Botha J, Witkowski ETF, Shackleton CMA (2004). Market profiles and trade in medicinal plants in the Lowveld, South Africa. *Environmental Conservation*.

[104] Lusa MG, Bona C (2011). Caracterização morfoanatômica e histoquímica de Cuphea carthagenensis (Jacq.) J.f. Macbr. (Lythraceae). *Acta Botanica Brasilica*.

[105] Lee C-L, Chen S-Y (2006). Classification of leaf images. *International Journal of Imaging Systems and Technology*.

[106] Wheeler QD (2004). Taxonomic triage and the poverty of phylogeny. *Philosophical Transactions of the Royal Society B*.

[107] Mati E, de Boer H (2011). Ethnobotany and trade of medicinal plants in the Qaysari Market, Kurdish Autonomous Region, Iraq. *Journal of Ethnopharmacology*.

[108] Hebert PDN, Cywinska A, Ball SL, de Waard JR (2003). Biological identifications through DNA barcodes. *Proceedings of the Royal Society B*.

[109] Mariot MP, Barbieri RL (2010). Divergência genética entre acessos de espinheira-santa (*Maytenus ilicifolia* Mart. ex Reissek e *M. aquifolium* Mart.) com base em caracteres morfológicos e fisiológicos. *A Revista Brasileira de Plantas Medicinais*.

[110] Coulaud-Cunha S, Oliveira RS, Waissmmann W (2004). Venda livre de *Sorocea bonplandii* Bailon como Espinheira-Santa no Município do Rio de Janeiro-RJ. *Revista Brasileira de Farmacognosia*.

[111] Diegues ACS, Arruda RSV (2001). *Saberes tradicionais e biodiversidade no Brasil*.

[112] Begossi A, Hanazaki N, Tamashiro JY (2002). Medicinal plants in the Atlantic Forest (Brazil): knowledge, use, and conservation. *Human Ecology*.

[113] Tabarelli M, Silva JMC, Araújo EL, Moura AN, Sampaio EVSB, Gestinari LMS, Carneiro JMT (2002). Áreas e ações prioritárias para a conservação, utilização sustentável e repartição de benefícios do bioma Caatinga. *Biodiversidade, conservação e uso sustentável da flora do Brasil*.

[114] Moerman DE, Estabrook GF (2003). Native Americans’ choice of species for medicinal use is dependent on plant family: confirmation with meta-significance analysis. *Journal of Ethnopharmacology*.

[115] Smelcerovic A, Spiteller M (2006). Phytochemical analysis of nine *Hypericum* L. species from Serbia and the F.Y.R. Macedonia. *Pharmazie*.

[116] Silva ML, Cechinel Filho V (2002). Plantas do gênero *Bauhinia*: composição química e potencial farmacológico. *Química Nova*.

[117] Guerra MP, Nodari RO, Simões CM (2004). Biodiversidade: aspectos biológicos, geográficos, legais e éticos. *Farmacognisia: da planta ao medicamento*.

[118] Brasil (2009). *Relação Nacional de Plantas Medicinais de Interesse ao SUS*.

[119] Albuquerque UP, Hanazaki N (2006). As pesquisas etnodirigidas na descoberta de novos fármacos de interesse médico e farmacêutico: fragilidades e pespectivas. *Revista Brasileira de Farmacognosia*.

[120] Vendruscolo GS, Mentz LA (2006). Levantamento etnobotânico das plantas utilizadas como medicinais por moradores do bairro Ponta Grossa, Porto Alegre, Rio Grande do Sul, Brasil. *Iheringia, Série Botânica*.

[121] de Almeida CDFCBR, Ramos MA, de Amorim ELC, de Albuquerque UP (2010). A comparison of knowledge about medicinal plants for three rural communities in the semi-arid region of northeast of Brazil. *Journal of Ethnopharmacology*.

[122] Diegues ACS (2000). *Etnoconservação—Novos Rumos para a Conservação da Natureza*.

